# Residue Interactions Guide Translational Diffusion of Proteins

**DOI:** 10.1021/acs.jpcb.4c06069

**Published:** 2025-02-25

**Authors:** Elham Fazelpour, Jennifer M. Haseleu, Christopher J. Fennell

**Affiliations:** † Department of Chemistry, Oklahoma State University, Stillwater, Oklahoma 74078, United States; ‡ School of Natural Sciences, Mathematics and Computing, St. Vincent College, Latrobe, Pennsylvania 15650, United States

## Abstract

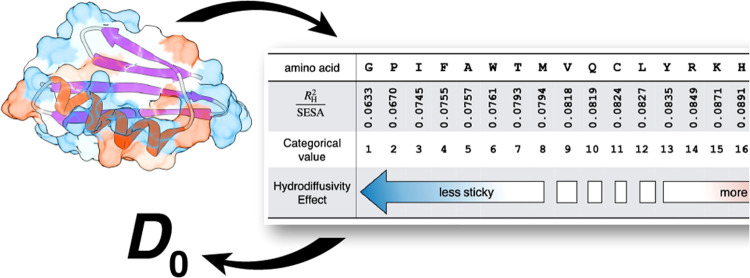

Diffusion at the molecular level involves random collisions between particles, the structure of local microscopic environments, and interactions among the molecules involved. Sampling all of these aspects, along with correcting for finite-size effects, can make the calculation of infinitely dilute diffusion coefficients computationally difficult. We present a new approach for estimating the translational diffusion coefficient of biomolecular structures by encapsulating these driving forces of diffusion through piecewise assembly of the component residues of the protein structure. By linking the local chemistry of a solvent-exposed patch of a molecule to its contribution to the overall hydrodynamic radius, an accurate prediction of the computationally and experimentally comparable diffusion coefficients can be constructed following a solvent-excluded surface area calculation. We demonstrate that the resulting predictions for diffusion coefficients from peptides through to protein structures are comparable to explicit molecular simulations and improve on statistical mass-based predictions, which tend to rely on limited training data. As this approach uses the chemical identity of molecular structures, we find that it is able to predict and identify differences in diffusivity for structures that would be indistinguishable by mass information alone.

## 1 Introduction

Diffusion in aqueous environments sets a fundamental limit on the kinetics of molecular processes involving peptide and protein structures. From cellular signaling to biotherapeutic action, the biomolecular structures involved need to translate and collide in order to initiate a response, often in fundamentally crowded environments.^1−3^ While the practical dynamics in real environments will be slower than the diffusion coefficient in pure water or an electrolyte solution, the diffusion coefficient of a single peptide or protein in a simple aqueous solution establishes the limit to the rate at which a biomolecule can passively traverse its environment. This infinitely dilute diffusion coefficient, *D*_0_, lacks the crowding and association effects that typically lead to a slowdown in particle translational dynamics.

Some of the early experimental efforts to determine the mass and shape of proteins used determinations of *D*_0_, by way of variations of Fick’s laws for diffusion of masses.^4^ This diffusion coefficient alongside other measurable properties of the aqueous solution can be input in the Svedberg equation to determine the mass of the primary solute.^5,6^ This work established the macromolecular identity of protein structures and helped pave the way for Théodor Svedberg receiving the Noble Prize in Chemistry in 1926. There are many modern techniques available to accurately determine protein masses, so many current processes run in the reverse direction by taking mass and structural information as an input to extract the diffusivity of a solute.^7−11^ Such methods often rely on statistical fitting to known *D*_0_ values, and this tends to make the resulting diffusivities more estimates rather than quantitative measurements.

Potentially better estimations of the diffusion coefficient, both infinitely dilute and environmentally specific, can be obtained from computational simulations. Such calculations can be computationally burdensome because sufficient sampling of the movement of large protein molecules, which are much larger than the surrounding solvent molecules, requires large system sizes and simulation times of many microseconds. Regardless of the specific system size chosen, the dynamics suffer from finite-size artifacts due to the use of periodic boundary conditions, and the diffusion coefficients calculated will be *apparent* diffusion coefficients, *D*_app_.^12^*D*_app_ can be converted to *D*_0_ by way of projecting to infinitely large systems by plotting the change in *D*_app_ with inverse simulation unit-cell length, 1/*L*.^13−15^ Accurate projections require many independent simulations with different *L*. Efforts to potentially avoid the computational cost of performing a systematic series of simulations have led to the introduction of more computationally convenient single-simulation finite-size corrections, the most popular being the proposed correction from Yeh and Hummer.^13,14^ Single-simulation corrections are extremely convenient; however, there are concerns about their validity as the correction magnitude may significantly vary depending on the system type and thermodynamic conditions.^16,17^

In this study, we are interested in exploring how the specific chemistry of biomolecules impacts their calculated translational diffusion coefficients. This starts with the accurate determination of the infinitely dilute diffusion coefficients for the standard amino acids that make up the bulk of the protein structure. Similar investigations have been done before,^18^ and our primary goal is to isolate the specific effect of amino acid chemical structure on the overall diffusion coefficient. The chemical identity of the individual amino acids determines protein hydrophilicity,^19^ and the relative favorability for hydration and solvent exposure is expected to impact protein diffusion in aqueous environments. From this systematic standpoint, we explore how the calculated *D*_0_ changes with increasing polymer chain length and assess the reliability of single-simulation finite-size corrections for calculating the diffusion coefficient of a peptide or protein. We further developed a rapid approach for predicting the diffusion coefficient from the chemical structure of a molecule exposed to the surrounding solvent.

## 2 Methods and Theory

The diffusion of spherical particles in a solvent with smooth flow is generally governed by the Stokes-Einstein equation,
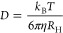
1Here η is the viscosity of the solvent, *T* is the temperature and *k*_B_ is the Boltzmann constant.^20^ While the hydrodynamic radius *R*_H_ of a spherical protein will be close to its geometric radius, the *R*_H_ will deviate from the geometric ideal due to the conformer shape and interactions with the environment. For example, water tightly bound to the protein solute surface will typically work to increase the hydrodynamic radius, decreasing the diffusion coefficient of the protein. Interactions with the environment do not need to be only with surrounding water. Interactions with ions, ligands, and other proteins will all typically work to increase the radius, so *R*_H_ is less a constant for a system and more a parameter that depends on the solution temperature, concentration, and other system state properties. This state dependence of *R*_H_ also extends to η, as state properties, such as the system temperature, will also influence the solvent viscosity.

In computer simulations of condensed phase systems, the translational and rotational dynamic behavior of solutes is dependent on the finite size of the simulation unit cell under periodic boundary conditions.^12,14,15,21,22^ With small unit cells, the apparent diffusion coefficient, *D*_app_, will be slower than that in simulations with large unit cells. Trends in *D*_app_ can be projected to infinitely large and dilute systems by linear plotting to where the inverse of the unit-cell lengths, *L*, from a series of simulations is equal to zero. This will give the point where *D*_app_ equals *D*_0_, but this unfortunately requires a series of simulations with differing unit-cell lengths. For cubic simulation unit cells, the *D*_app_ can be treated as an *L*-dependent function,
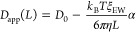
2where *k*_B_ is the Boltzmann constant, *T* is the simulation temperature, *L* is the simulation box edge length, η is the solvent viscosity, ξ_EW_ ≈ 2.837298 is a unitless cubic lattice self term,^23^ and α is an empirical parameter.^15^ This α parameter was introduced by Yeh and Hummer to earlier correction formulas to account for suspected differences due to solute–solvent interactions present in charged RNA solute simulations that are not present when the solute is neutral or a point perturbation.^13,15^

In the case that the *D*_0_ computed from molecular simulations is to be compared to experimental values, it is important to note that the solvent in simulations is not a perfect representation of water at the chosen state point. For example, the TIP3P water model is notably less viscous than real water.^14,24^ The disparities between simulation and experiment can be corrected by multiplying the simulation *D*_0_ by the ratio of simulation and experimental viscosity,
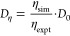
3The shear viscosity of water is unaffected by simulation system size but is sensitive to temperature, pressure, salt concentration, and long-range interaction accumulation treatments for electrostatics.^14,24,25^ Therefore, for each unique state point and system composition, η_sim_ should be calculated for the ratio with the experimental system analog.

### 2.1 Computational Methods

#### 2.1.1 Peptide and Protein Simulations

Molecular dynamics simulations of all aqueous peptide and protein systems were performed using GROMACS 5.1.5.^26−29^ All models were represented with the Amber99SB-ILDN force field, with TIP3P used for the water model and any neutralizing counterions using the associated Amber force field ion types.^30−34^

Monopeptide and decapeptide systems were prepared in an extended conformation using the Antechamber tool in the AmberTools package, with ACE and NME capping groups to neutralize the zwitterionic character of the backbone.^35,36^ In addition to monopeptides of each of the standard amino acids in the protonation state at pH 7, an *N*-methylacetamide solute was crafted manually using the topologies of the ACE and NME capping groups. Structures and topologies of all solutes were converted to GROMACS format using the ACPYPE Python script.^37^ For protein structures, the starting conformers of the molecules were downloaded from the Protein Data Bank (PDB) and conversion to topology files was done using the PDB2GMX tool available in the GROMACS package.

Simulation configurations were built with the insertion of single-capped monomer amino acids in cubic unit cells with edge lengths of 3.0, 3.5, 4.0, 4.5, and 5.0 nm. Larger peptide simulations involved solute insertion into cubic unit cells with lengths ranging from 5.0 to 7.0 nm in steps of 0.5 nm, while protein simulation cubic unit-cell lengths ranged from 6.0 to 8.0 nm in steps of 0.5 nm. If necessary, these systems were neutralized with the appropriate number of sodium or chloride counterions inserted in random locations in the unit cells. All systems were then solvated with TIP3P water with a nominal density near 1000 kg m^–3^.^32^ For each simulation size and type, 10 distinct starting configurations were generated and seeded with random velocities from a Maxwell–Boltzmann distribution following 1000 kJ mol^–1^ nm^–1^ force-converged steepest descent minimization. All were equilibrated for 150 ps of isotropic constant pressure (1 atm) and constant-temperature (310.15 K) dynamics, with the Parrinello–Rahman barostat and v-rescale thermostat using time constants of 10 and 1 ps, respectively.^38,39^ The leapfrog technique with a time step of 3 fs was used to integrate the equations of motion, LINCS to constrain hydrogen atom bond vibrations, and SETTLE to keep TIP3P water molecules rigid.^40,41^ Note that 3 fs is a longer time step than is common practice for aqueous molecular simulations. This time step was chosen to efficiently access long-time dynamics states after performing a successful energy conservation assessment in *NVE* simulations of a test system at the target temperatures. We further validated the use of this time step through the calculation of identical *D*_0_ values for ALA monopeptide using both a 2 and 3 fs time step. For long-ranged electrostatics correction, a smooth particle-mesh Ewald with a real space cutoff of 1.0 nm, spline order of 4, and energy tolerance of 10^–5^ was applied.^42^ Long-range energy and pressure corrections were applied for Lennard-Jones interactions also cutoff at 1.0 nm.^43^

Following *NPT* equilibration, 100 ns molecular dynamics trajectories were recorded for each simulation. Given that there are 10 independent simulations for each system size and composition, each cubic biomolecule simulation data point involved 1 μs of aggregate sampling. The mean-square displacement, MSD, was used to compute the apparent diffusion coefficient as a function of time,
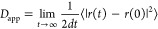
4

Here, *d* is the desired dimension (1, 2, or 3), and the displacement, *r*(*t*) – *r*(0), is taken across the dimensions of interest. The most linear portion of the MSD curve as a function of time, ranging from 30 to 500 ps for monopeptides and from 30 to 3000 ps for proteins, was used for estimating the slope via the least-squares fitting method. The value of *D*_*app*_ was then calculated as one-sixth of the obtained slope given the three-dimensional simulation.

**Figure 1:**
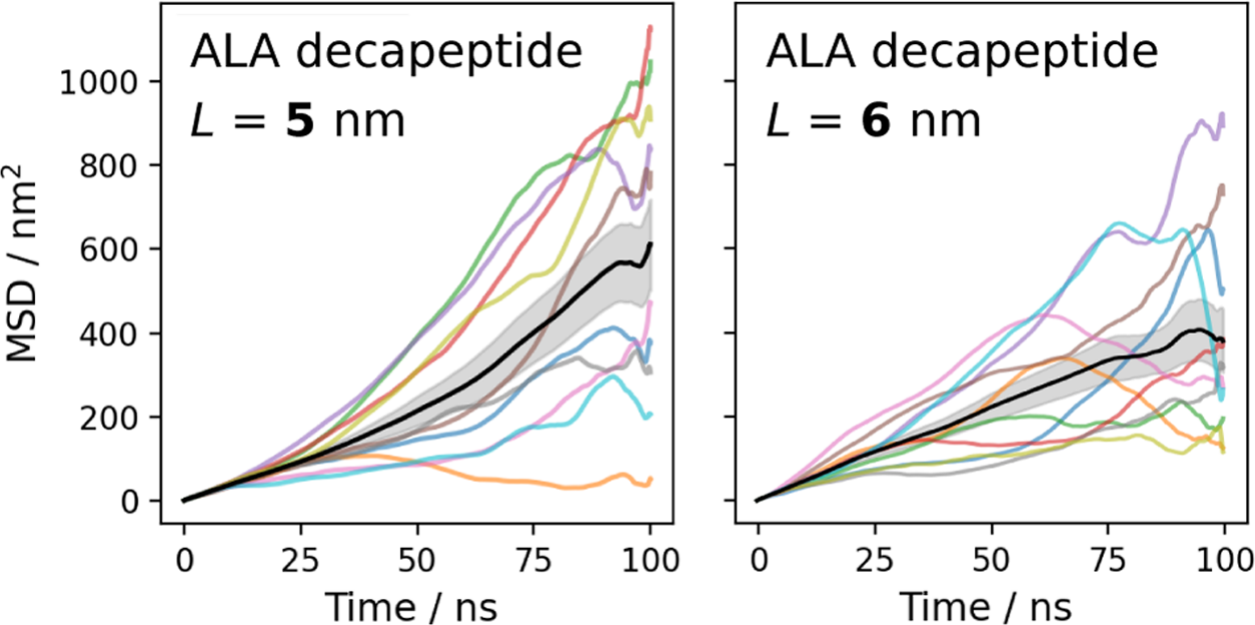
MSD curves for single ALA decapeptides in the smallest (*L* = 5 nm on the left panel) and middle (*L* = 6 nm on the right panel) simulation unit cells are linear below 10 ns time intervals. The colored lines show the 10 independent MSD particle traces, and these traces are averaged to give the black line with a gray error envelope.

Example MSD curves are shown in Figure 1 for simulations of capped ALA decapeptide in the smallest simulation unit cell (*L* = 5 nm) and middle-sized simulation unit cell (*L* = 6 nm). The 10 colored lines in each plot are the individual solute particle traces for the 10 separate simulations, which are averaged to give the black line with the error envelope shown with gray shading. Because of the limited long-time-interval statistics, there is significant variability in the solute MSD values. For example, the orange particle trace in the *L* = 5 nm plot appears to return to near the starting position after about 80 ns, while the green and red particle traces continually drift to more distant positions as a function of time. A key point to note in these plots is the individual traces begin to noticeably deviate from the average MSD curve between 5 to 10 ns. This is the reason for limiting the linear regression for determination of the slope to short-time intervals, less than 3 ns. While subtle, the slope of the larger system size average MSD curve is steeper in this short-time regime than the average MSD curve in the smaller system. This is a visual demonstration of the increasing *D*_app_ as a function of the increasing system size. Each calculated *D*_0_ from molecular dynamics involves 50 of these simulation MSD traces, 10 independent simulations over 5 different system sizes.

#### 2.1.2 Shear Viscosity of Water

The shear viscosity of the TIP3P water model was calculated using MD simulation with 900 molecules in a cubic box with an edge length of 3.0 nm.^32^ The shear viscosity of a liquid is related to the fluctuations of the off-diagonal elements of the pressure or stress tensor, according to the Navier–Stokes equation,
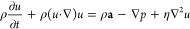
5In this equation, **a** is the external force per unit mass and volume. This approach is employed in the periodic perturbation method, and at each time step of the simulation, the periodic external force is imposed on molecules.^44^ Further details on this technique can be found in the Supporting Information (Section S1).

A series of MD simulations ranging from 273.15 to 323.15 K with increments of 5 K were performed using GROMACS 5.1.5 to address the temperature dependency of shear viscosity values for use in diffusion value predictions comparable with experiments.^29,45^ Additionally, the impact of salinity on viscosity was explored by calculations involving various concentrations of NaCl in TIP3P water. The same system setup described above was used with nominal NaCl concentrations of 0.125, 0.25, 0.5, 1.0, and 2.0 M at 293.15 K.

For each series, 10 distinct starting configurations were generated and seeded with random velocities from a Maxwell–Boltzmann distribution at the target temperature following 1000 kJ mol^–1^ nm^–1^ force-converged steepest descent minimization. All were equilibrated for 1 ns of *NVT* ensemble sampling followed by 1 ns *NPT* ensemble sampling with the Berendsen thermostat and barostat using time constants of 1 ps.^46^ This *NPT* ensemble simulation step is necessary to determine the density of the system, which is used in the final canonical ensemble sampling simulations. The leapfrog technique with a time step of 2 fs was used to integrate the equations of motion, and SETTLE to keep TIP3P water rigid.^40^ All molecular dynamics options were the same as in the previously detailed peptide and protein simulations, with the exception of the use of a 0.9 nm particle interaction cutoff to better match with published simulation options.^44^

Following a 1 ns *NVT* equilibration with a periodic acceleration value, *A* of 0.005 nm ps^–2^ at the target density from the *NPT* simulation, 20 ns *NVT* ensemble molecular dynamics trajectories were accumulated for each system. As each state point involved 10 independent simulations, this resulted in 200 ns of aggregate sampling.

### 2.2 Residue-Based Analysis

**Figure 2:**
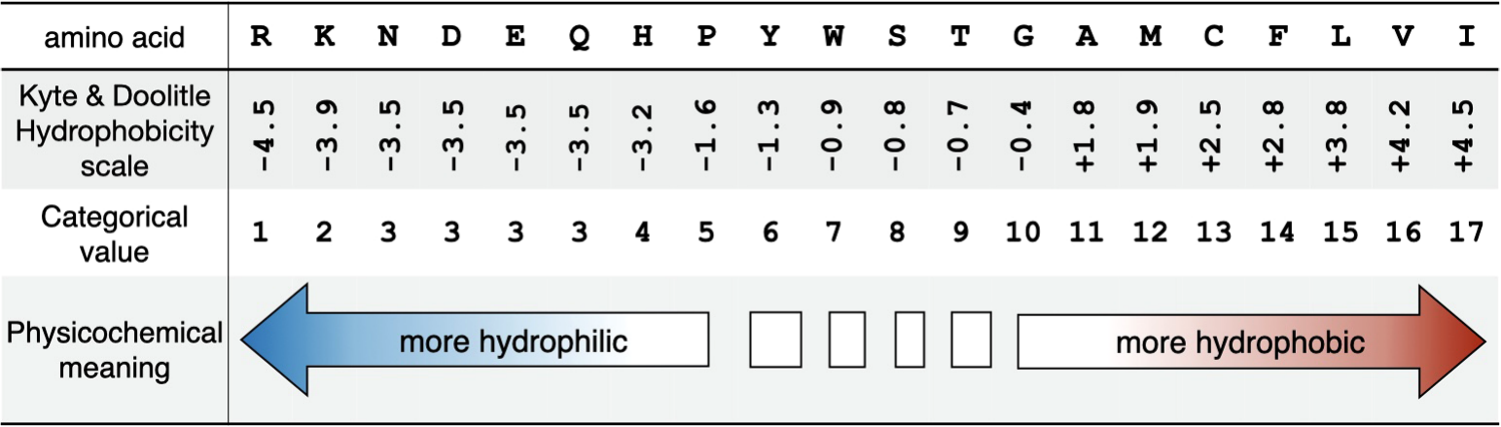
The hydropathy index introduced by Kyte and Doolittle uses both computational and experimental data on the water vapor transfer free energies of proteins with different amino acid side chains.

Similar to how a hydropathy index like the Kyte and Doolittle scale (Figure 2) rank associates the hydration favorability to the chemical identity of the individual amino acids,^19^ we attempted to isolate the effect of the unique chemistry of each residue on the translational diffusion coefficient for an overall biomolecular structure. The goal of this work is to develop and provide a simple tool for estimating the expected *D*_0_ from molecular simulations. In this way, we could potentially provide finite-size corrected and experimentally comparable diffusion coefficients that encode the specifics of a given solute’s environment, be it a unique water model, salinity, temperature, pH, etc.

To build an applicable hydrodiffusivity index, the Stokes–Einstein equation can be rearranged to focus on the hydrodynamic radius of each of the amino acid residues calculated from the finite-size corrected diffusion coefficients,
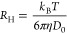
6The *R*_H_ is a quantity related to the geometrical radius of a spherical particle, though it is altered by the specific interactions that the solute has with its environment as previously discussed. Assembling an *R*_H_ from the solute components would allow for the prediction of a simulation diffusion coefficient. The surface area has been shown to have a strong geometric relationship with the *R*_H_ of protein structures,^25^ so basing the contributions to the radius from the surface exposure of the solvent-exposed residues should result in a relevant overall hydrodynamic radius.

Assembling an *R*_H_ of a general protein from its surface area patches starts with a surface area calculation, one that includes the surface area contributions from the types of amino acids. MSMS 2.6.1 was used to determine the solvent-excluded surface area (SESA) of individual capped amino acids and the *N*-methylacetamide (NMA) molecule, using a 0.15 nm probe radius.^47−49^ Unitless hydrodiffusivity parameters, *C*_solute_, were computed for each of these solutes via,
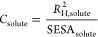
7To determine the uncapped hydrodiffusivity parameter for each of the monomeric amino acids, *R*_H,solute_^2^ = *R*_H,cappedAA_^2^ – *R*_H,NMA_^2^, and SESA_solute_ = SESA_cappedAA_ – SESA_NMA_. The hydrodynamic radius of a full protein can be additively accumulated by summing the product of this hydrodiffusivity parameter and the SESA of the respective amino acid types over the whole protein,
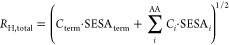
8where AA is the set of 20 standard amino acids, *C*_*i*_ is the uncapped hydrodiffusivity parameter of the *i*-th amino acid type, and SESA_i_ is the accumulated solvent-excluded surface area of all atoms belonging to each *i*-th amino acid type. The C- and N-terminus contributions to the *R*_H_ come from the term before the summation, where SESA_term_ is the solvent-excluded surface area of an NMA molecule. *C*_term_ depends on whether the sequence is capped with ACE and NME groups or if the biomolecule has a zwitterionic backbone. If it is capped, *C*_term_ is the hydrodiffusivity parameter computed from the NMA solute simulation. If it is zwitterionic and has formal charges that strongly interact with the surrounding solvent, *C*_term_ is approximated with the average of the ASP and LYS monopeptide hydrodiffusivity parameters, because of their formal charge nature at pH 7. The average *R*_H_ of uncapped ASP and LYS is very similar to the overall average *R*_H_ of zwitterionic amino acids presented in other work, supporting its use as a quantitative proxy for the zwitterionic terminus contribution.^18^ The diffusion coefficient of the peptide or protein can then be computed by using this *R*_H,total_ in place of the hydrodynamic radius in the Stokes–Einstein equation (eq 1). This process is henceforth referred to as Residue Interaction Diffusion Estimation, RIDE, and enables rapid estimation of *D*_0_ values from molecular simulation and can be used to estimate *D*_η_ values from PDB structures for comparison with experimental diffusion coefficients.^6,8,25^

A RIDE calculation requires two input files: (1) a PDB file containing a protein’s structural information and (2) an area file that contains the solvent-excluded surface area of each atom in this PDB file. For estimations of peptide and protein *D*_0_ values with RIDE, the MSMS program was used to generate the per-atom area file, again with a 0.15 nm probe radius to be consistent with the probe size used in generating the hydrodiffusivity parameters. In the cases of prediction of *D*_0_ from a molecular dynamics trajectory, the RIDE calculation used the centroid conformer from the trajectory, following an RMSD clustering of the ensemble. The chosen temperature and viscosity values used in the calculation come from the respective simulation temperature and simulation salt concentration.

## 3 Results and Discussion

### 3.1 Shear Viscosity of TIP3P Water Is Needed at Corresponding Temperatures for Finite-Size Hydrodynamics Corrections

The viscosity of the environment of a solute is inversely proportional to the diffusion coefficient of the solutes under laminar flow conditions (eq 1). It is also a critical component in hydrodynamic finite-size corrections for aqueous environments, though it has been demonstrated to not be impacted by finite-size effects itself.^13,15^ Given its utility and use in potential *D*_η_ estimations, we explored both the temperature dependence of the viscosity and the viscosity of aqueous solutions as a function of NaCl concentration.

Table 1 provides the result for viscosity as a function of the temperature. As noted by other studies on the dynamics of TIP3P water, it is known to be a low viscosity model relative to the experimental liquid,^14,24,50^ and these results overlap within error to values computed by others at comparable temperatures.^14,51^ In conjunction with this low calculated viscosity, the change in viscosity is quite flat in the plotting of this data (see Figure S1). This indicates that any viscosity correction of a diffusion coefficient for a solute present in TIP3P should consider the specific temperature of the system to best compensate for these changes. In the amino acid diffusion calculations, the standard body temperature of 37 °C (310.15 K) was chosen, and a viscosity of 0.275 ± 0.004 mPa·s was used in the correction of diffusion coefficients for finite-size effects with these solutes.

**Table 1:** Calculated Viscosity (in mPa·s with Error of the Last Digit in Parentheses) of TIP3P Water as a Function of Temperature with Comparison to Experimental Values Computed from a Fit to Data in ref (52)

*T* (K)	TIP3P	expt.
273.15	0.445(5)	1.793
278.15	0.412(7)	1.518
283.15	0.388(3)	1.307
288.15	0.355(4)	1.137
293.15	0.337(3)	1.002
298.15	0.319(4)	0.890
303.15	0.299(5)	0.798
308.15	0.283(4)	0.719
313.15	0.273(6)	0.653
318.15	0.254(3)	0.596
323.15	0.244(2)	0.547

While TIP3P is not a strongly cohesive water model by itself, changes in viscosity with the presence of ions more accurately follow the behavior seen in experimental solution viscosity. While the viscosity is roughly 3 times lower than the experiment in pure water simulations (Table 1), the change in solution viscosity tracks well with the experiment up to a nominal 1.0 M concentration of NaCl (see Figure S2). This indicates that accurate predictions of simulation diffusion coefficients could potentially come by way of experimental quantities for changes in viscosity with a change in salinity.

### 3.2 Finite-Size Diffusion Correction Factor α Values Are Not Uniform

In an attempt to isolate the effect of individual residue interactions on the translational diffusion coefficient, the finite-size corrected diffusion coefficients were calculated for the 20 standard amino acids at their pH 7 protonation states in TIP3P water. Table 2 shows the calculated *D*_0_ values at 310.15 K for single amino acids with the backbone capped with acetyl and *N*-methyl groups. The capping groups increase the mass of monopeptides over uncapped peptides with zwitterionic backbones, and this along with the greater temperature is why the *D*_0_ values from this work differ in places from those calculated by Zhang et al.^18^Table S2 more clearly shows the slowdown due to the mass of the capping groups as *D*_0_ values at 298.15 K are presented alongside values computed with the OPC water model.^53^ The peptides in Table 2 are sorted by molar mass, and while the *D*_0_ values generally decrease with increasing molar mass, there are deviations. For example, while PHE weighs over 30 g/mol more than ASP, their capped monopeptides have diffusion coefficients that are nearly the same. This occurs because water binds more tightly to the deprotonated carboxylic acid of ASP than to the phenyl ring of PHE, resulting in ASP having a greater *effective* mass. In this way, stronger interactions with the environment will lead to increased hydrodynamic radii and proportionally slower diffusion.

**Table 2:** Calculated *D*_0_ Values in 10^–5^ cm^2^ s^–1^ and α Finite-Size Correction Parameters for the Acetyl and *N*-Methyl Capped Mono- and Decapeptides at Infinite Dilution in TIP3P Water at 310.15 K, with Error in the Last Digit in Parentheses

	monopeptide		decapeptide	
	*D*_0_	α	*D*_0_	α
GLY	2. 68(8)	1.07(2)	1. 40(4)	0.874(9)
ALA	2. 44(6)	1.01(2)	1. 27(5)	0.91(1)
SER	2. 23(7)	0.81(2)	1. 28(4)	0.96(1)
PRO	2. 39(2)	1.009(4)	1. 16(6)	0.84(2)
VAL	2. 19(3)	0.891(8)	1. 15(1)	0.843(3)
THR	2. 25(3)	0.999(8)	1. 18(5)	0.86(2)
CYS	2. 28(6)	0.85(1)	1. 24(5)	0.82(1)
ILE	2. 15(2)	0.954(5)	1. 09(3)	0.73(3)
LEU	2. 07(5)	0.87(1)	1. 11(4)	0.81(1)
ASP	2. 01(7)	0.91(2)	1. 03(3)	0.881(8)
ASN	2. 10(3)	0.848(9)	1. 12(1)	0.821(3)
GLU	1. 93(5)	0.95(2)	1. 00(5)	0.95(1)
GLN	2. 08(3)	1.00(1)	1. 06(3)	0.826(8)
LYS	1. 93(5)	0.91(2)	0. 93(1)	0.811(4)
MET	2. 07(3)	0.85(1)	1. 14(1)	0.872(3)
HIS	1. 99(2)	0.909(6)	1. 09(3)	0.960(7)
PHE	2. 03(4)	0.892(5)	1. 08(7)	0.824(8)
ARG	1. 87(2)	0.92(1)	0. 88(2)	0.89(2)
TYR	1. 93(3)	0.913(6)	1. 03(2)	0.707(6)
TRP	1. 91(2)	0.892(9)	1. 07(3)	0.915(5)
avg.^a^	2.13(5)	0.92(2)	1.12(3)	0.86(2)

a Std. error from column values in parentheses.

Somewhat concerning in these calculations are the variable slopes observed for *D*_app_ versus 1/*L* for extraction of the finite-size α correction factor calculated from these simulations. The original periodic diffusion coefficient correction derived by Dünweg and Kremer involved neutral polymer solutes, and the introduction of the empirical α parameter by Yeh and Hummer was justified given their study of charged polymeric solutes. With solutes closer to neutral point perturbations, one should expect an α correction factor of 1. In simulations of K^+^ ions and trinucleotide RNA (−2 charge), Yeh and Hummer determined correction factors of 0.88 and 0.76 respectively. This presumably indicates that greater absolute formal charges and stronger solute–solvent interactions will lead to decreasing α. The results in Table 2 do show a decrease in α with stronger solute–solvent interactions, although the regularity of this decrease is not clear. The smallest α belongs to SER, but THR can also hydrogen bond, and it has a value near 1. Neutral VAL has α = 0.89, while formally charged LYS has α ≈ 1. This lack of uniformity makes applying a correction factor more of a speculative endeavor rather than a systematic predictive process.

As expected, the decapeptide results in Table 2 show simulation *D*_0_ values that are slower than those of monopeptides. The average slowdown indicates the decapeptides diffuse at nearly half the rate of the monopeptides, and this mainly comes from the decapeptides having greater relative surface areas, volumes, and masses. In addition to the decreased *D*_0_ values, we also observe a decrease in the α finite-size correction parameter. A greater deviation from the ideal value of 1 is understandable given the greater deviation from an ideal periodic point defect with a larger peptide size. Unfortunately, there does not appear to be a uniform trend in the systematic deviations. While LYS shows a significant decrease in α in going from LYS to LYS_10_ (0.91–0.81), GLU_10_ is unchanged from GLU, and the SER_10_ α actually increases to 0.96 over the SER value of 0.81. This variability leads to upward of 30% errors in the applied finite-size correction term (note the variation in ILE and ARG α values) for these small peptides. Using a finite-size correction to estimate *D*_0_ from *D*_app_ will typically provide a more accurate value, but α values do not appear to be generally transferable. The most accurate calculations of *D*_0_ will come from the intercept of a 1/*L* regression from a series of system sizes.

### 3.3 Simulation Diffusion Coefficients Can Be Assembled to Predict Peptide and Protein Diffusion

From the calculated diffusion coefficients of the individual amino acids, it is possible to predict the translational diffusion coefficient of peptides or proteins, without needing to know a specific finite-size correction term. To enable this, we constructed a hydrodiffusivity scale that can be used to additively assemble a hydrodynamic radius from the unique surface exposure of a peptide or protein. In this scale, hydrodiffusivity coefficients (*R*^2^/SESA) were computed for each of the amino acids as described in Section 2.2. For this process, a *D*_0_ of 3.32(9) × 10^–5^ cm^2^/s was calculated and used for *N*-methylacetamide. Additionally, these coefficients are determined at a physiological temperature, 310.15 K, with the corresponding viscosity of TIP3P water as these were the conditions of the specific molecular simulations of the monopeptides.

**Figure 3:**
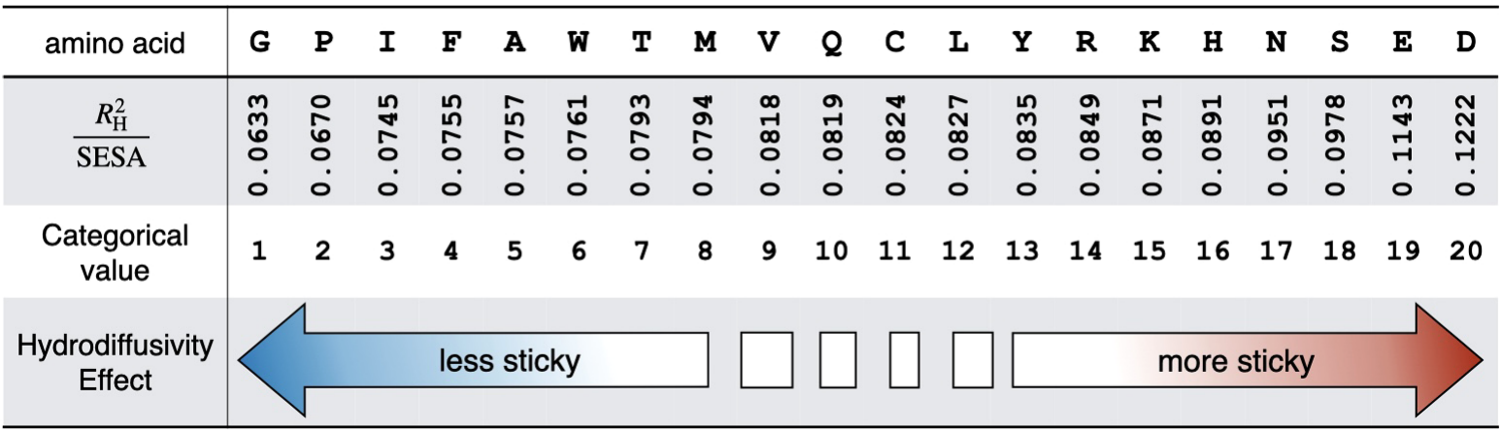
Hydrodiffusivity scale encodes the effect that residue contact with water has on the hydrodynamic radius of the peptide and protein structures. Amino acids with weak interactions with water, like isoleucine (I) and glycine (G), contribute less to the *R*_H_ than residues like serine (S) and aspartate (D), which can interact with hydrogen bonding and ion-dipole forces, respectively.

Figure 3 shows the set of hydrodiffusivity coefficients for the monopeptides. Similar values for the amino acids from the decapeptides are available in the associated Supporting Information, Table S3. The resulting scale conveys the effect that a surface patch of a given amino acid has on the molecule’s hydrodynamic radius. Amino acids that cannot form hydrogen bonds or ion-dipole interactions with the surrounding water contribute much less than those residues that can form strong interactions. For example, each equivalently sized surface area patch from glycine (G) on a protein will contribute roughly half that of an aspartate (D) toward the *R*_H_^2^ of the molecule. In principle, the charged and polar amino acids cause the greatest hydrodynamic drag when in contact with the surrounding aqueous solvent. This indicates complete mutation of the surface of a protein from glycine-like to aspartate-like chemistry will cause a given protein to diffuse upward twice as slowly in water. There is some anomalous sorting in this scale, as leucine (L) appears significantly stickier than isoleucine (I), this despite their similar chemistry and size. This difference does go away when considering decapeptides, which involve more SESA averaging and factor secondary structure propensity into the values.

To test the additivity of the RIDE process, the finite-size corrected *D*_0_ value was calculated for a poly-alanine peptide series up to the ALA_6_ hexapeptide. The RIDE predictions in Table 3 are shown for both the monopeptide hydrodiffusivity coefficients, RIDE(M), and the decapeptide hydrodiffusivity coefficients, RIDE(D). The calculated *D*_0_ decreases with an increasing chain length because of the greater size and solvent contact area of the peptides. The RIDE(M) and RIDE(D) models reproduce this *D*_0_ value series well, often within the simulation error. In all cases, RIDE(D) produces slightly larger predictions than RIDE(M), this is because the hydrodiffusivity coefficient for monopeptide ALA is 0.0757 while for decapeptide ALA is 0.0718. The smaller coefficient in RIDE(D) leads to a smaller hydrodynamic radius in the additive assembly and a correspondingly greater *D*_0_ estimation. The smaller RIDE(D) coefficients and correspondingly faster *D*_0_ predictions are systematic for all amino acids. This comes about because decapeptides can sample conformations that have a secondary structure, hiding primarily polar backbone groups from the surrounding water. Less polar group contact with the solvent will lower the hydrodiffusivity coefficients, as seen in Figure 3.

**Table 3:** Comparison of *D*_0_ Values in 10^–5^ cm^2^ s^–1^ for Poly-alanine Peptide with Different Chain-Lengths Calculated from Simulations in TIP3P Water at 310.15 K versus RIDE(M) and RIDE(D) Predictions

name	*D*_0_	*D*_RIDE(M)_	*D*_RIDE(D)_
ALA	2.44(5)	2.47	2.48
ALA_2_	2.01(4)	2.06	2.07
ALA_3_	1.73(3)	1.80	1.81
ALA_4_	1.61(2)	1.62	1.63
ALA_5_	1.49(2)	1.50	1.51
ALA_6_	1.44(3)	1.38	1.40

### 3.4 Predictions Using Residue Interactions and Secondary Structure Propensity Can Outperform Mass Relations and Explicit Finite-Size Corrections

Accurate calculation of the infinitely dilute diffusion coefficient of a peptide or a protein is computationally costly. It requires simulations of the target solute with multiple unit-cell sizes and tens of microseconds of aggregate dynamics sampling. This is why the finite-size correction popularized by Yeh and Hummer is generally appealing. A single very long trajectory is still needed to determine an apparent diffusion coefficient to potentially correct. A much more rapid *D*_0_ determination can come from a simple mass relation.^7,54^ In the assembly process of RIDE, a surface area calculation of a single structure could be used to arrive at a *D*_0_, making it significantly more rapid to calculate than a finite-size corrected *D*_0_ from an explicit computer simulation.

**Figure 4:**
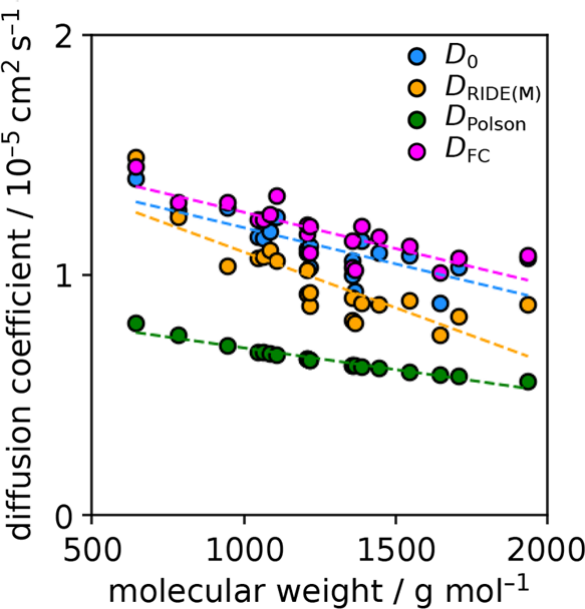
Without an α parameter, finite-size corrections (FC) tend to predict faster diffusion coefficients than the explicit *D*_0_ of decapeptides in TIP3P water at 310.15 K. The Polson mass relation significantly under-predicts *D*_0_, and RIDE(M) more modestly under-predicts *D*_0_. The linear trend lines are a visual guide.

Figure 4 plots the explicit simulation-determined *D*_0_ value alongside finite-size corrected (FC), mass relation (Polson), and RIDE(M) predictions for the decapeptide series of amino acids. Most noticeable are the *D*_Polson_ predictions that underestimate the diffusion coefficients. These mass relation predictions use a *C* coefficient derived from an ultracentrifugation-determined value from hemoglobin, and the predictions here include a correction to scale to the simulation temperature and a final correction for the difference in viscosity for TIP3P and experimental water at the simulation temperature,
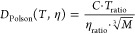
9where *T*_ratio_ is the target temperature in Kelvin over 293.15 K and η_ratio_ is the viscosity of TIP3P solvent at the target temperature over the experimental viscosity of water at 293.15 K. It is apparent that all the *D*_Polson_ values are systematically slow and nonvariable. Even choosing to use a *C* coefficient from a smaller cyclic decapeptide like Gramicidin S or Tyrocidine will be insufficient, as this would shift the final prediction by little more than 5%. Additionally, the mass relation prediction trend is generally too shallow with respect to changes in molecular weight. This comes from the cube root term in the denominator of the mass relation function, connecting the mass to the diffusion coefficient by way of the molecular volume.^7,25^ If this molecular volume-based restriction is relaxed to instead be an expansion of the mass to different powers of *M* rather than one-third, one could potentially more freely fit to biomolecules over a variety of masses, this with some loss in physical meaning for the mass relation terms.^55^ We recently explored mass relations that are fit and connect instead through the surface area, and this provided a foundation for the assembly process of the RIDE technique.^25^

The other insights from the results shown in Figure 4 are that FC predictions without the known α parameter tend to systematically predict faster diffusion coefficients than the full explicit *D*_0_. This is because the α parameters are generally less than 1 and scale the prediction down. If the α parameter was accurately known, the FC prediction would be identical to the explicitly calculated *D*_0_. RIDE(M) predictions using simple helical structures are much more accurate than mass relation values, though they generally under-predict the actual *D*_0_.

**Figure 5:**
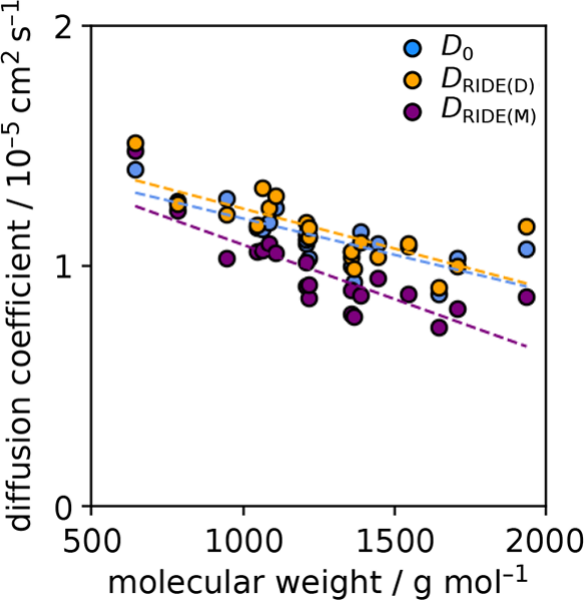
Inclusion of the secondary structure propensity in hydrodiffusivity coefficients leads to near quantitative *D*_0_ estimations. While RIDE(M) tends to under-predict the dynamics of decapeptides, RIDE(D) can rapidly provide predictions equivalent to those from the application of a finite-size correction (Figure 4) on explicit TIP3P water simulations at 310.15 K.

The underprediction of diffusion coefficients from RIDE(M) is due to the fact that the monopeptide backbone is going to be solvent exposed. The amide groups on the backbone can hydrogen bond with the surrounding water, leading to a larger contribution to the *R*_H_. Figure 5 shows what happens if the secondary structure propensity is factored into the hydrodiffusivity coefficients. Expectedly, the RIDE(D) predictions are in much better agreement with the explicit *D*_0_ values. The slope of a linear trendline is also much closer to that of the explicit calculations, and the mean signed error (MSE) for the set (0.018 × 10^–5^ cm^2^ s^–1^ or 1.6%) is the same as the average error for the *D*_0_ values and an improvement over the −15.2% RIDE(M) MSE. The RIDE(D) predictions of *D*_0_ are only moderately faster than the periodic boundary condition corrected *D*, and this comes from the use of perfectly helical compact states. Making a RIDE(D) prediction using a more generally representative centroid structure from an explicit simulation trajectory would likely further refine the prediction, though at the computational cost of performing a simulation that produces a structure representative of the equilibrium ensemble.

The *D*_FC_ values use an α value of 1 and predict the average decapeptide *D*_0_ 5.7% too fast. One could consider using the average α computed from all of the decapeptides shown in Table 2, 0.86(2), as a placeholder value for a finite-size correction parameter for any biomolecule built from the 20 standard amino acids. This average value is coincidentally similar to the value of 0.88 extracted by Yeh and Hummer for diffusion of a K^+^ ion and closer to 1 than the 0.76 from the trinucleotide RNA strand. This limited comparison again indicates that α is dependent on the size of the solute, which is consistent with the mono- to decapeptide average trend in Table 2, and the presence of a radius term of the expansion of the α parameter performed by Yeh and Hummer to unit-cell length, *L*, and spherical solute geometric radius, *R*, function.^14^ Knowing and applying this average α for the decapeptides in a finite-size correction of the smallest-size decapeptide MD simulation (*L* = 5 nm) *D*_app_ value does result in a near-perfect prediction of *D*_0_. The MSE prediction improves from 5.7 to −0.2%, outperforming the RIDE(D) prediction of 1.6%. This indicates that one could potentially use RIDE(D) to estimate an average protein α value (like the 0.86 value for the decapeptides) to help determine a *D*_0_ from a single *D*_app_ value computed from long-time MD trajectory of a system with a small *L*.

### 3.5 Salt Concentration Can Be Accurately Treated as a Change in Solvent Viscosity

The translational dynamics of peptides and proteins are intimately connected to the state of the environment. As the temperature increases, we should see a corresponding and proportional increase in the infinitely dilute diffusion coefficient via eq 1. Similarly, the viscosity is inversely proportional to the infinitely dilute diffusion coefficient. In our calculation of simulation viscosity, we observed a trend of change in viscosity as a function of salt concentration that is in line with experimental measurements up to a roughly 1 M concentration. This viscosity trend was mapped into the RIDE process as a salt concentration input.

**Figure 6:**
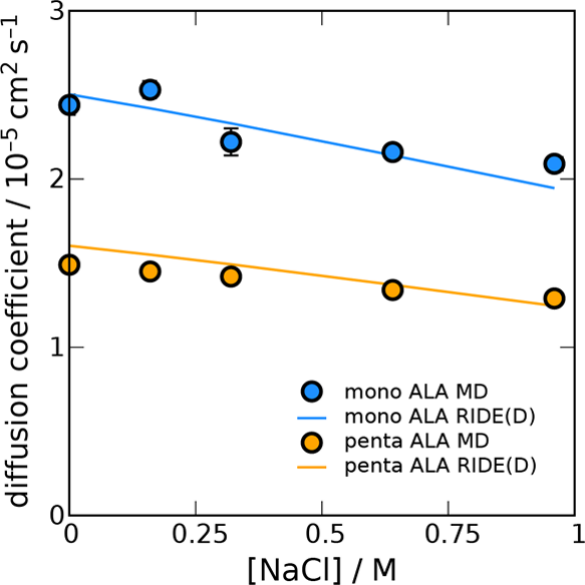
Salt effects primarily materialize as changes in the solvent viscosity. Applying the TIP3P solution viscosity trend as a function of salt concentration in RIDE(D) (lines) reproduces the results for the ALA monopeptide and pentapeptide in explicit TIP3P water simulations (points) at 310.15 K.

Figure 6 shows the results for comparisons of both capped alanine mono- and pentapeptide *D*_0_ as a function of the NaCl concentration. Molecular dynamics calculations show a roughly linearly decreasing trend over this nominal salt concentration range. As with the decapeptide predictions, the RIDE(D) calculations used perfectly helical structures, only now with a salt concentration perturbation of the TIP3P viscosity. The RIDE(D) results reproduce this decreasing trend quite accurately for both the mono- and pentapeptides. Alanine peptide is a rather clean test case, as we do not expect significant specific ion effects such as ion–peptide association to discretely alter the solute dynamics. This may not be the case for charged peptides, and future consideration of the role of ion-pairing may need to be included in such systems.

### 3.6 Considering Structure and Interactions Can Lead to More Accurate Estimation of Diffusion

**Figure 7:**
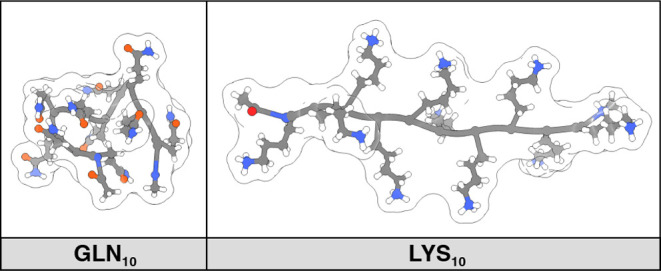
LYS_10_ (right panel) adopts conformations more extended than those of GLN_10_ (left panel) in explicit molecular simulations, leading to increased solvent exposure and a correspondingly slower diffusion coefficient. These decapeptide structures are the central conformers from clustering analysis of their respective trajectory data.

Relying solely on molecular weight to estimate explicit simulation diffusion coefficients does not fully capture the trend and variability in dynamics, as shown clearly in Figure 4. Each of the amino acids will interact with the surrounding water in a unique way based on its local structure. For example, GLN_10_ and LYS_10_ have similar molecular weights and this results in near identical diffusion coefficient via a mass relation. However, a comparative analysis of their trajectory ensembles reveals distinct conformational preferences (Figure 7). LYS_10_ primarily adopts a more extended structure, leading to increased solvent interactions and a slower diffusion coefficient, while GLN_10_ adopts more compact conformations, resulting in reduced solvent exposure and a faster diffusion rate.

To further investigate the potential effect that peptide conformations have on the diffusion coefficient, we performed a DSSP analysis on the simulation trajectories of the decapeptides to assess the secondary structure population over the course of the molecular simulations.^56^ The results of this analysis are shown in Figure S3. The results give some insight into the conformers adopted by the decapeptides over the course of an equilibrium MD trajectory used to determine the apparent diffusion coefficient. Populations support the observed representative conformers shown in Figure 7, with LYS_10_ showing hardly any bend/turn population in the ensemble, reflecting primarily extended conformers in the ensemble. About 30% of the GLN_10_ population consists of bends/turns, resulting in a compact overall state, one that is similar to that seen for about half of the decapeptide populations. The helical structures used in calculating the RIDE(D) *D*_0_ values are certainly more ordered than the typical conformers adopted by all the decapeptides, aside from PRO_10_ which is primarily found in a polyproline helix conformation. More disordered conformers would likely show increased solvent exposure of the backbone, leading to slightly slower predictions from RIDE(D). It should be noted that prolate or oblate distortion of conformers in an ensemble will always lead to an increase in SESA (see discussion in Supporting Information), so RIDE will naturally incorporate some shape distortion impact on translational diffusion. There are other approaches that have been developed that consider structural and shape contributions to the diffusion of biomolecules.^10,11,57−59^ The original aspherical extensions of the Stokes–Einstein equation incorporate the full mobility tensor of a rigid body, helping consider how diffusion along a long-axis direction tends to be faster than along shorter axes that present a greater area profile to the surrounding solvent.^10,11^ One example tool that takes advantage of the shape and particulate structure of macromolecules is HYDROPRO, which builds residue- or atomic-level structural models of a given protein for computing hydrodynamic properties.^58,59^ For making accurate connections to the diffusion coefficients of biomolecular structures, the size of the particulate component beads is scaled to fit known protein diffusion coefficients. Once fit, the connected network or shell of bead representation of similarly sized related proteins tends to be well-reproduced.^59^

The peptide and protein structures explored in this study are all smaller than the proteins considered in the development of HYDROPRO, indicating that we should not expect an accurate reproduction of *D*_0_ computed by MD simulations for decapeptide structures. Using the residue-level bead model with the recommended bead radius and viscosity of water at the target temperature for all the decapeptide structures, we obtained an average *D*_0_ = 0.34 × 10^–5^ cm^2^ s^–1^, which is more than 3 times slower than the MD-calculated average of 1.12 × 10^–5^ cm^2^ s^–1^ and 2 times slower than the temperature and viscosity corrected mass relation. The HYDROPRO atomistic-level shell model performs similarly with *D*_0_ = 0.35 × 10^–5^ cm^2^ s^–1^. The bead sizes for these representations can be refitted to more accurately reproduce the surface interaction effect on the diffusion for these smaller systems, though it is clear that the bead size is not a universally applicable physical constant.

By factoring in interactions, explicit structure propensity, and finite-size corrections from polypeptide simulations, the hydrodiffusivity coefficients used in RIDE(D) can enable simple, rapid, and accurate predictions of simulation *D*_0_ values, and these can be scaled to determine *D*_η_ values comparable to experimental quantities. To better emphasize the importance of interactions beyond just mass, we computed explicit *D*_0_ values for protein G mutants that have very high sequence and mass similarity, but differing folds.^60,61^ Diffusion coefficients determined from a mass relation should be nearly identical for the mutant pairs, but the *D*_0_ values should differ. For this comparison, two primary series of protein G binding domains, namely G_A_ and G_B_ (PDB IDs: 2FS1 and 1PGB), were chosen alongside metamorphic proteins G_A_95, and G_B_95 (PDB IDs: 2KDL and 2KDM) that were designed to possess 95% sequence identity while exhibiting different tertiary structures.^60,61^

**Figure 8:**
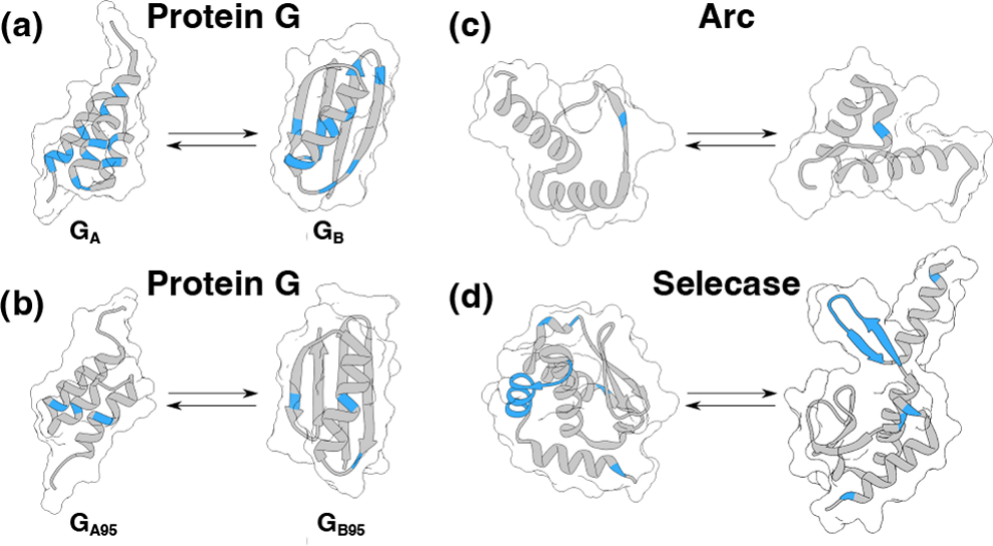
Metamorphic proteins have similar sequences but distinctly different structures. In the four metamorphic subunit protein pairs shown, sites of mutation that lead to the change in structure are colored blue, and regions of sequence similarity are gray. PDB IDs for the displayed structures are (a) 2FS1 and 1PGB, (b) 2KDL and 2KDM, (c) 1BDT and 1QTG, and (d) 4QHF and 4QHH.

The calculated *D*_0_ values for this set of four 56 residue count structures alongside mass relation and RIDE(D) predictions, with a zwitterionic backbone contribution, are shown in Table 4. The G_A_ and G_A95_ proteins are compact 3-helix bundles while the G_B_ and G_B95_ proteins have a 4-stranded sheet and single helix (see Figure 8). The latter proteins are less compact and exhibit an average of roughly 200 Å^2^ larger SESAs, and they somewhat counterintuitively diffuse more rapidly despite this increased solvent contact. As with the decapeptides, the mass relation results tend to be slower than MD simulations, though this deviation is likely due to inadequate temperature and viscosity corrections in eq 9. The RIDE(D) results tend to be a bit faster than the MD calculations, but they often overlap with the reported error of the MD simulation *D*_0_ values. The key finding in these results is the distinct difference in the predicted diffusion coefficients for the G_A_ and G_B_ domains with RIDE(D). The differences are around half that seen in explicit simulations, and these differences come from the specifics of the solute–solvent contact interactions and the change in SESA from the structural change. For a method that increases the hydrodynamic radius of a protein with increasing contact, the G_B_ proteins would incorrectly be predicted to diffuse more slowly if there was no consideration of the specific interactions of the amino acid residues with the surrounding solvent.

**Table 4:** Comparison of *D*_0_ Values in 10^–5^ cm^2^ s^–1^ of Wild-Type and Mutated G Protein (Masses Shown in kg mol^–1^) from Simulations in TIP3P Water at 310.15 K, the Polson Mass Relation, and RIDE(D)

	MW	*D*_0_	*D*_Polson_	*D*_RIDE(D)_
G_A_	6.14	0.52(6)	0.388	0.565
G_B_	6.19	0.59(8)	0.387	0.608
G_A95_	6.31	0.46(6)	0.385	0.562
G_B95_	6.35	0.62(8)	0.384	0.587

Not included in the RIDE(D) calculations is knowledge of the overall aspect ratio and flexibility of the protein. A particle with a nonspherical overall shape will potentially have different rates of diffusion along the principle axes of protein, and differences in eccentricity can lead to a greater difference in *D*_0_ values seen between the G_A_ and G_B_ proteins. Additionally, the RIDE(D) predictions are based on the first of the experimental PDB structures, and simulation structures will adopt different, potentially more expanded, configurations.

To further show how sequence similarity does not necessarily dictate equivalent hydrodynamic behavior, we selected additional pairings of metamorphic and chameleonic proteins that have been subject to or undergone evolutionary changes that specifically alter their structures.^62−66^ In many cases, these proteins are part of larger complexes or have a change in oligomeric state, but the interest here is simply on the change in structure, despite their sequence similarity. The mutations that distinguish these sequence pairs are minimal and do not alter the net charges of the pairings. This means that the molecular weights of the protein pairs will be similar and there are few other distinguishing qualities beyond shape and structure. Table 5 shows predicted experimental *D*_η_ values from a mass relation and RIDE(D) for the Arc and Selecase protein mutant pairs, illustrated in Figure 8c,d.

**Table 5:** Comparison of Experimental Diffusion Coefficient, *D*_η_, Values in 10^–5^ cm^2^ s^–1^ of Arc Switch (1BDT and 1QTG) and Selecase (4QHF and 4QHH) Proteins Using the Polson Mass Relation Method and *D*_η_ from RIDE(D) at 293.15 K

PDB	MW	# res	chg	*D*_Polson_	*D*_RIDE(D)_
1BDT	6.14	52	+4	0.150	0.148
1QTG	6.23	53	+4	0.149	0.151
4QHF	12.94	109	+6	0.117	0.119
4QHH	12.81	108	+6	0.117	0.108

In both the Arc and Selecase pair cases, a mass relation shows nearly identical predicted experimental diffusion coefficients. The Polson mass relation is designed to focus on predictions of the diffusion coefficient in pure water at 293.15 K, so no temperature or viscosity corrections need to be applied in order to make comparisons. There is actually strong agreement between RIDE(D) and the Hemoglobin-based mass relation for expected experimental diffusion coefficients, and this indicates that the applied temperature and viscosity corrections in eq 9 are of limited flexibility for reproducing the *D*_0_ from molecular dynamics simulations at varied temperatures. The main differences are a subtle variation in the heavier Arc mutant, being expected to diffuse very slightly faster than the lighter Arc mutant. This counterintuitive behavior is amplified in the case of the larger Selecase protein pair. Both of these cases involve a deformation of the structure rather than a dramatic reorganization of the tertiary structure seen in the protein G cases, and the signal is expectedly more subtle. These predictions indicate that we should expect potentially measurable variation in the protein dynamics for mutations that are critical points for dictating the local and extended secondary structures of a protein.

A potentially intriguing aspect of the protein G, Arc, and Selecase mutant pairings is the absence of previously known experimental diffusion information. The predictions here are made solely on knowledge-based diffusion behavior. Shape and interactions, used in RIDE(D), better predict anomalous simulation-based *D*_0_ behavior than mass. However, mass relations, like the Polson approach, are simple and can provide convincing experimental estimations, as they are statistically fit to experimental values. The resulting experimental prediction comparisons shown in Table 5 show a surprising consistency between the mass relation and RIDE(D), despite RIDE(D) not using any experimental diffusion data. The fact that RIDE(D) uses the structural and chemical identity of a protein while matching the prediction capability of a statistically fit mass relation potentially provides researchers with new insight into how the sequence of a biomolecule connects to its dynamical properties.

## 4 Conclusion

Accurately determining infinitely dilute diffusion coefficients of biomolecules through computational methods can be difficult. Projecting to the infinitely dilute state requires multiple increasingly large simulations, each typically having a significant computational cost. Using a finite-size correction requires only a single system size; however, the uncertainty in the magnitude of the finite-size correction factor leads to an uncertainty in the calculated diffusion coefficient.

In this study, we demonstrate an approach that involves additive assembly of a peptide or protein’s hydrodynamic radius that considers the interactions at the interface between the solute and the surrounding solvent. This process can enable rapid estimation of a finite-size corrected diffusion coefficient for a molecule composed of standard amino acids. By factoring in changes in solvent viscosity via temperature and ionic strength, we can make estimations for unique simulation environments and experimental systems. The RIDE technique is able to arrive at accurate estimations of infinitely dilute diffusion coefficients, and it is a rather simple platform that can be modified with increasingly detailed considerations to overcome limitations. For example, monopeptide diffusion is strongly influenced by backbone exposure to the solvent, and extracting hydrodiffusivity coefficients from polypeptides incorporates secondary structure propensity, which will mediate this overemphasis of the importance of protein backbone exposure on dynamics.

Interactions between protein residues and the surrounding water play a critical role in biomolecular function and dynamics. Accounting for these interactions in a detailed manner via the amount of solvent contact and the nature of the local forces can lead to improvements in the analysis and predictive understanding of the natural collective motions of the peptide and protein structures.

## Supporting Information Available

Additional method details, tabulated data from plots, and source code for two versions of the RIDE program. (PDF)
