# Supplementary material for: Residue Interactions Guide Translational Diffusion of Proteins
Source: J Phys Chem B. 2025 Feb 25;129(9):2493–504. doi: 10.1021/acs.jpcb.4c06069 (PMC11891898; doi:10.1021/acs.jpcb.4c06069)
Supplement: Supplementary file 1 — jp4c06069_si_001.pdf [file jp4c06069_si_001.pdf]

Residue interactions guide translational diffusion of proteins

Elham Fazelpour,[†] Jennifer M. Haseleu,[†,‡] and Christopher J. Fennell[*,†]

†*Department of Chemistry, Oklahoma State University, Stillwater, Oklahoma 74078, USA*

‡*School of Natural Sciences, Mathematics and Computing, St. Vincent College, Latrobe, Pennsylvania 15650, USA*

E-mail: christopher.fennell@okstate.edu

Supporting Information Overview

This document contains data and results for the viscosity of TIP3P water molecules, an investigation of the connection between molecular surface area and asphericity, results from secondary structure ensemble analysis of decapeptide MD simulations, and translational diffusion coefficient calculations of and predictions for single amino acids as well decapeptide sequences of amino acids presented in the accompanying manuscript. Example text of the RIDE Perl code (used in manuscript predictions) and a version converted to Python are placed at the end for convenient example tool use and testing.

S1 Calculation of shear viscosity of TIP3P water

In this study, Navier-Stokes equation was used to calculate the viscosity of water.

$$\rho\frac{\partial u}{\partial t} + \rho(u\cdot\nabla)u = \rho\,\mathbf{a} - \nabla p + \eta\nabla^2 u \tag{1}$$

The periodic external force $\boldsymbol{a}$ is imposed on molecules in a way in a way that a_y and a_z are zero and a_x is a function of z.[1,2] As a result, u_y and u_z are equal to zero. Equation 1 reduces to

$$\rho \frac{\partial u_x(z)}{\partial t} = \rho\, a_x(z) + \eta \frac{\partial^2 u_x(z)}{\partial z^2} \tag{2}$$

At steady-state the solution is given by

$$a_x(z) + \frac{\eta}{\rho}\frac{\partial^2 u_x(z)}{\partial z^2} = 0 \tag{3}$$

Given we simulated a periodic system, the acceleration and velocity profiles should be periodic as well. A simple cosine function can smooth the acceleration profile to obtain smooth profiles for local shear rate and velocity.

$$F_{i,x} = m_i a_x(z) = m_i A \cos(kz) \tag{4}$$

Where $k = \frac{2\pi}{l_z}$ with l_z being the height of the box and A is the applied acceleration. The generated velocity profile at steady state ($u_z(x) = 0$, $t = 0$) is

$$u_x(z) = \nu(1 - e^{-t/\tau_r}) \cos(kz) \tag{5}$$

$$\nu = A\frac{\rho}{\eta k^2} \tag{6}$$

where τ_r is the macroscopic relaxation time of the liquid, which is given by

$$\tau_r = \frac{\rho}{\eta k^2} \tag{7}$$

The viscosity of the liquid can be estimated by calculating the averaged ν during an MD simulation using the following equation:

$$\eta = \frac{A}{\nu}\frac{\rho}{k^2} \tag{8}$$

Table S1: Comparison of experimental values of viscosity of water and the simulated viscosity of TIP3P water as a function of temperature. The corrected viscosity of TIP3P water was calculated using the ration between the Exp. and Simulated values at 298.15 (K), respectively and applying that on the simulated data.

Temperature (K)	Exp. (mPa.s)	TIP3P (mPa.s)	Corrected TIP3P (mPa.s)
273.15	1.793	0.445(5)	1.244
278.15	1.518	0.412(7)	1.147
283.15	1.307	0.388(3)	1.085
285.15	1.137	0.355(4)	0.993
293.15	1.002	0.337(3)	0.941
298.15	0.890	0.319(4)	0.890
303.15	0.798	0.299(5)	0.836
308.15	0.719	0.283(4)	0.789
310.15	0.691	0.275(4)	0.768
313.15	0.653	0.273(6)	0.763
318.15	0.596	0.254(3)	0.711
323.15	0.547	0.244(2)	0.682

S2 Asphericity impact on solvent excluded surface area

The shape of a molecule can have a significant impact on its translational diffusion. This has been formally explored by Brenner back in the 1960s via generalization of the Stokes-Einstein relation for diffusion to distorted spheroids.[3,4] The RIDE technique detailed in the main text uses the standard Stokes-Einstein relation and simply poses the determination of a diffusion coefficient by way of a R_{H} that encodes unique chemical aspects of the system's biomolecular structure. By using the spherical system approximation, RIDE does not include any aspherical considerations that one might focus on in traditional hydrodynamic theory. RIDE does utilize the impact of SESA on the diffusivity, as the R_{H} comes from the modulated SESA by way of the surface area of a sphere. Any distortion of a sphere along two of the principle axes that maintains the same volume will *always* result in an increase in surface area. Connecting to a R_{H} by way of the surface area, this with constant particle volume, will implicitly encode some of the impact of asphericity on the diffusion coefficient.

To show this asphericity connection, we first set a constraint of the volume of a sphere and the volume of an ellipsoid,

$$\frac{4\pi r^3}{3} = \frac{4\pi abc}{3}, \tag{9}$$

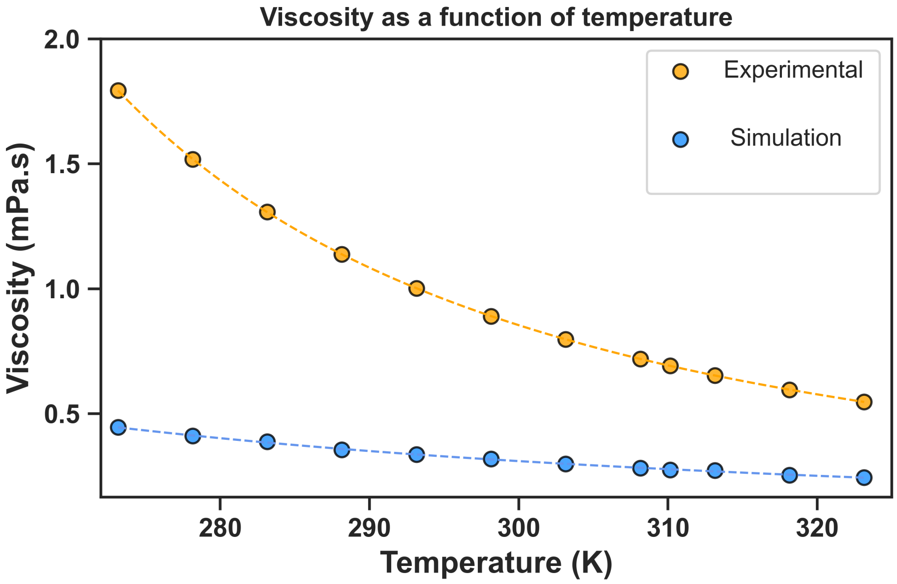

(a)

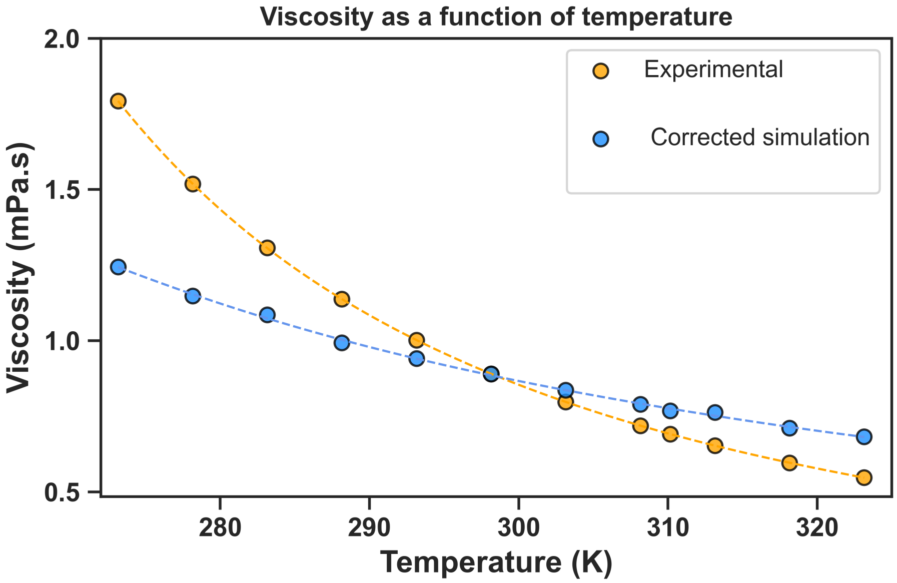

(b)

Figure S1: In plot (a) the orange dots illustrate the experimental values of viscosity, and the blue dots represent the simulated viscosity of TIP3P water as a function of temperature. (b) shows the applied correction on the simulated viscosity of TIP3P water and how the experiment and the corrected-simulation overlap.

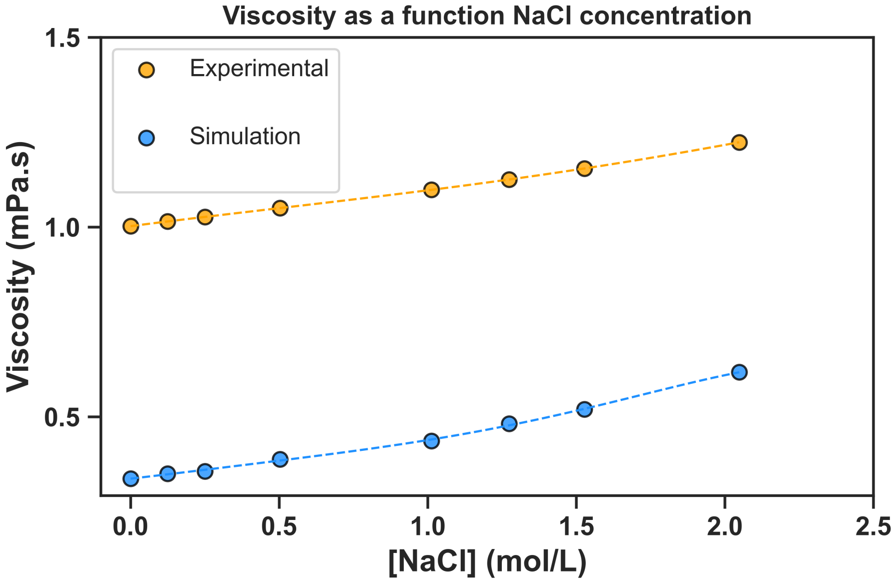

(a)

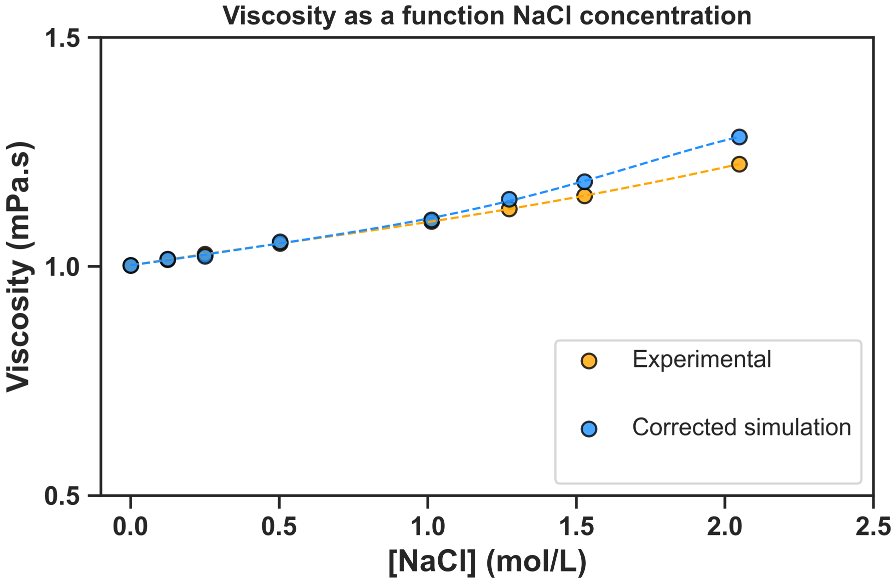

(b)

Figure S2: (a) demonstrates the experimental and simulated values of viscosity as a function NaCl concentration. (b) shows the applied correction on the simulated viscosity of TIP3P water and how the experiment and the corrected-simulation overlap.

where r is the radius of a sphere and a, b, and c are the three principal axes of the spheroid. In a sphere, $a = b = c = r$, so $r^3 = abc$. The surface area for an ellipse, S, can be approximated by,

$$S \approx 4\pi \left(\frac{(a \cdot b)^{8/5} + (a \cdot c)^{8/5} + (b \cdot a)^{8/5}}{3} \right)^{5/8}. \tag{10}$$

We can rewrite this S equation as a function of r and x, where r is the radius for a spherical object and x is the absolute deformation of one of the radius along one of the principle axes. Because of the constant volume constraint, this becomes,

$$S \approx 4\pi \left(\frac{(r^2 + r \cdot x)^{8/5} + \left(\frac{r^3}{r+x}\right)^{8/5} + (r^2)^{8/5}}{3} \right)^{5/8}, \tag{11}$$

where one of the other three principle axes counters the distortion to maintain this constraint. This function can be plotted as a function of both r and x, but this is more generally and simply visualized by selecting a constant radial, r, value and plotting this as a function of just x.

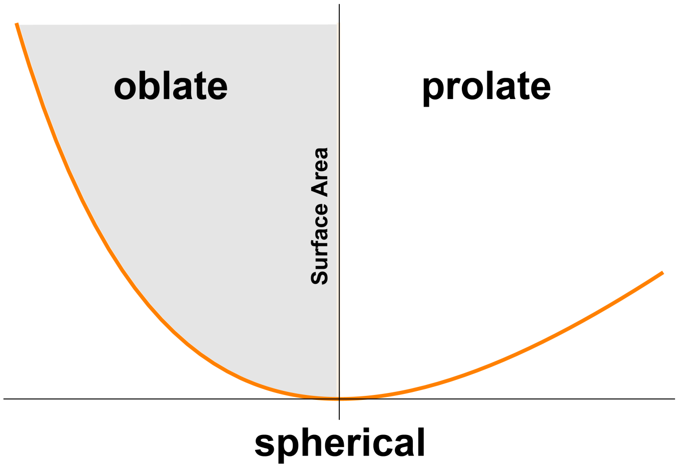

Figure S3: The surface area of a distorted sphere (center line) always increases with distortion of a principle axis under the constraint of constant spheroid volume.

Figure S3 shows a plot of Equation 11 where r is set to be a constant value. For elongation of a principle axis, the surface area shows a gradual increase with increasing distortion and formation of a prolate style spheroid. For compression of a principle axis, this is an oblate type spheroid distortion, and we see a similar increase in surface area. In principle, these coupled distortions

give effectively similar spheroid objects, but the compression distortion is limited to no more than the magnitude of the radius of the original sphere. Both types of distortion will leave us with an infinitely long (and infinitely thin) ribbon with width of r.

S3 Secondary structure analysis of decapeptide systems

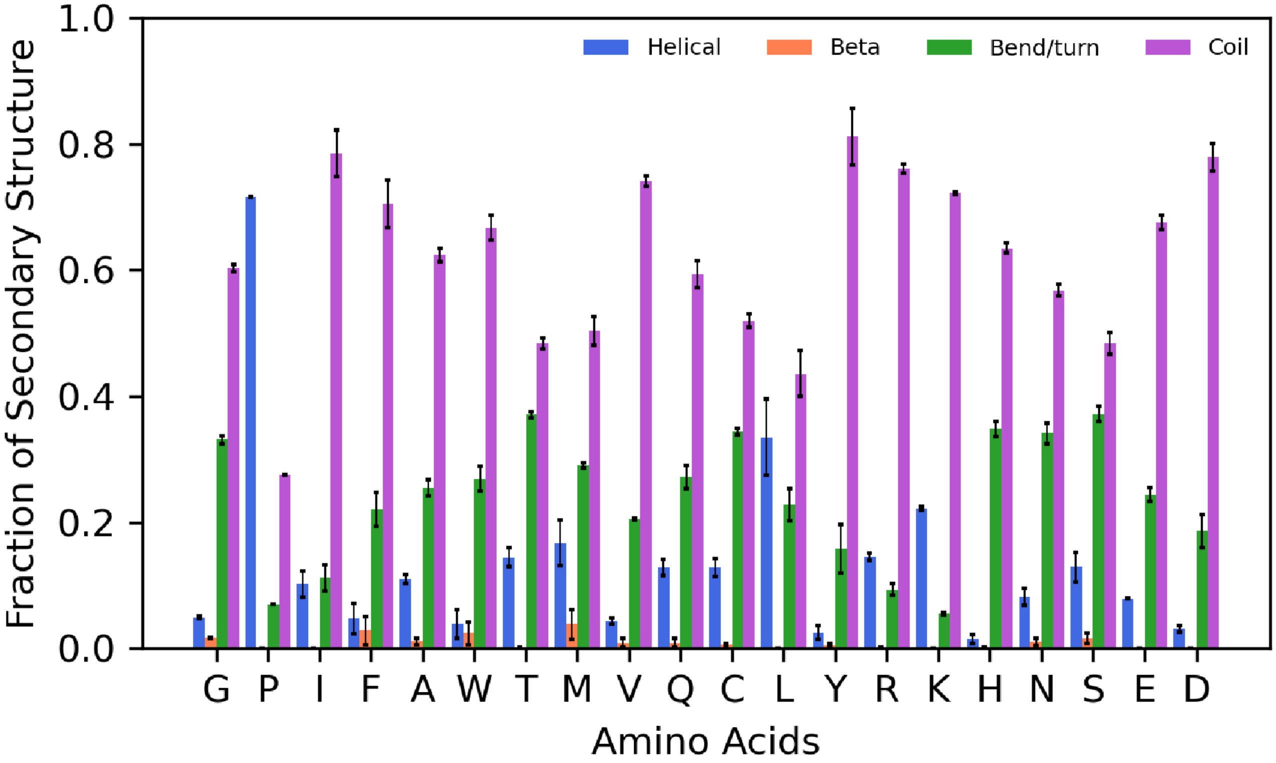

Figure S4: Secondary structure populations show distinct conformational behavior in molecular simulations of decapeptides of the 20 standard amino acids. Of the amino acids secondary structure populations, PRO tends to form polyproline helix structures primarily, while LEU also shows a high helix propensity. Most of the decapeptides adopt coil conformers, which are generally more exposed and extended than turns and helical structures.

The DSSP tool was used to analyze conformers from regularly sampled trajectories of the largest system size molecular dynamics simulations for all the decapeptides studied in the main manuscript.[5] The fraction of helical, beta, bend/turn, and coil secondary structure were binned and the resulting distributions (with error) are shown in Figure S4. The key takeaways from this analysis are that rigid PRO tends to primarily sample extended polyproline helix conformations while all other amino acids will favor a more disordered coil structure. LYS nearly has equal populations of helix and coil structures. Larger populations of bend states are indicative of more compact

ensembles because the side-chains are able to interact with the backbone and other side-chains following a bend.

In principle, aside from PRO, the ensembles are disordered over primarily helix conformers. Such distortions will tend to increase the average SESA of the representative structures of ensembles. In RIDE, this should translate to slower overall diffusion coefficients when using rigorous representative state structures.

Table S2: Comparison of calculated translational diffusion coefficients (in 10^{-5} cm^2 s^{-1}) of amino acids with experimental values at infinite dilution.

Amino acid	This work TIP3P (310.15 K)	TIP3P (298.15 K)	OPC (298.15 K)	TIP3P[a] (298.15 K)	Expt[b] (298.15 K)
ARG	1.86(3)	1.55(3)	0.83(3)	1.96	0.55
TRP	1.91(2)	1.59(2)	0.80(2)	1.83	-
LYS	1.93(4)	1.60(4)	0.85(1)	1.75	0.53
GLU	1.93(7)	1.60(7)	0.86(11)	1.83	0.62
TYR	1.93(2)	1.60(2)	0.87(3)	1.82	-
HIS	1.99(5)	1.65(5)	0.93(2)	2.00	0.56
ASP	2.01(3)	1.66(3)	0.87(5)	1.89	0.65
PHE	2.03(5)	1.69(5)	0.86(4)	1.89	0.60
LEU	2.07(3)	1.72(3)	0.85(4)	2.17	-
MET	2.07(2)	1.72(2)	0.90(1)	2.22	0.64
GLN	2.08(5)	1.73(5)	0.86(3)	1.98	-
ASN	2.09(3)	1.74(3)	0.89(2)	2.16	0.66
ILE	2.15(2)	1.79(2)	0.87(2)	2.07	0.62
VAL	2.18(3)	1.81(3)	0.92(4)	1.87	-
SER	2.23(1)	1.85(1)	1.05(5)	2.20	0.71
THR	2.25(7)	1.87(7)	0.94(2)	2.30	-
CYS	2.27(3)	1.89(3)	1.01(3)	2.33	0.69
PRO	2.38(6)	1.98(6)	1.01(1)	-	-
ALA	2.44(6)	2.02(6)	0.95(4)	2.40	0.74
GLY	2.68(8)	2.22(8)	0.93(7)	2.76	-

[a] adapted from Ref. 6
[b] adapted from Ref. 7

Table S3: Comparison of diffusion coefficient values (in 10^{-5} cm^2 s^{-1}) of capped deca-peptides using D_0 calculated from simulations in TIP3P water at 310.15 K and RIDE prediction method. (Note: 1 Da = 1 g mol^{-1})

Name	MW (Da)	D_0	$\frac{R_H^2}{SESA}$[a]	D_{Polson}	$D_{\mathrm{RIDE(M)}}$	D_{FC}[a]
GLY_{10}	643.61	1.40(4)	0.0599	0.85	1.48	1.45
ALA_{10}	783.87	1.27(5)	0.0718	0.79	1.23	1.30
SER_{10}	943.87	1.28(4)	0.0677	0.74	1.03	1.30
PRO_{10}	1044.25	1.16(6)	0.0537	0.72	1.06	1.23
VAL_{10}	1064.40	1.15(1)	0.0504	0.72	1.06	1.23
THR_{10}	1084.13	1.18(5)	0.0595	0.71	1.09	1.25
CYS_{10}	1104.52	1.24(5)	0.0520	0.71	1.05	1.33
ILE_{10}	1204.67	1.09(3)	0.0530	0.69	1.01	1.17
LEU_{10}	1204.67	1.11(4)	0.0521	0.69	0.91	1.21
ASP_{10}	1213.89	1.03(3)	0.0699	0.69	0.87	1.09
ASN_{10}	1214.12	1.12(1)	0.0575	0.69	0.92	1.20
GLU_{10}	1354.15	1.00(5)	0.0661	0.66	0.80	1.03
GLN_{10}	1354.39	1.06(3)	0.0573	0.66	0.90	1.14
LYS_{10}	1364.90	0.93(1)	0.0543	0.66	0.79	1.02
MET_{10}	1385.05	1.14(1)	0.0490	0.66	0.88	1.20
HIS_{10}	1444.49	1.09(3)	0.0528	0.65	0.85	1.16
PHE_{10}	1544.83	1.08(7)	0.0477	0.63	0.88	1.12
ARG_{10}	1645.03	0.88(2)	0.0554	0.61	0.74	1.01
TYR_{10}	1704.83	1.03(2)	0.0555	0.61	0.82	1.07
TRP_{10}	1935.19	1.07(3)	0.0408	0.58	0.87	1.08

[a] Yeh-Hummer method[8]

Table S4: Prediction of Diffusion coefficients of capped α-helical decapeptides where hydrodiffusivity values of monomer ($D_{RIDE(M)}$) vs. decamers ($D_{RIDE(D)}$) are incorporated in RIDE prediction method at 310.15 K. (D values in 10^{-5} cm^2 s^{-1})

Name	MW (Da)	D_0	$D_{\mathrm{RIDE(M)}}$	$D_{\mathrm{RIDE(D)}}$
GLY_{10}	643.61	1.40(4)	1.48	1.49
ALA_{10}	783.87	1.27(5)	1.23	1.24
SER_{10}	943.87	1.28(4)	1.03	1.20
PRO_{10}	1044.25	1.16(6)	1.06	1.15
VAL_{10}	1064.40	1.15(1)	1.06	1.31
THR_{10}	1084.13	1.18(5)	1.09	1.22
CYS_{10}	1104.52	1.24(5)	1.05	1.27
ILE_{10}	1204.67	1.09(3)	1.01	1.16
LEU_{10}	1204.67	1.11(4)	0.91	1.11
ASP_{10}	1213.89	1.03(3)	0.87	1.10
ASN_{10}	1214.12	1.12(1)	0.92	1.14
GLU_{10}	1354.15	1.00(5)	0.80	1.02
GLN_{10}	1354.39	1.06(3)	0.90	1.04
LYS_{10}	1364.90	0.93(1)	0.79	0.97
MET_{10}	1385.05	1.14(1)	0.88	1.08
HIS_{10}	1444.49	1.09(3)	0.85	1.07
PHE_{10}	1544.83	1.08(7)	0.88	1.08
ARG_{10}	1645.03	0.88(2)	0.74	0.90
TYR_{10}	1704.83	1.03(2)	0.82	0.98
TRP_{10}	1935.19	1.07(3)	0.87	1.14

Table S5: D_0 in 10^{-5} cm^2 s^{-1} of ALA_1 and ALA_5 in TIP3P water at 310.15 K as a function of nominal NaCl concentration alongside RIDE(D) predictions.

[NaCl]	$ALA_1 D_0$	$ALA_1 D_{\mathrm{RIDE(D)}}$	$ALA_5 D_0$	$ALA_5 D_{\mathrm{RIDE(D)}}$
0.00	2.44(6)	2.503	1.49(2)	1.603
0.16	2.53(5)	2.420	1.45(4)	1.550
0.32	2.22(8)	2.331	1.42(2)	1.493
0.64	2.16(2)	2.140	1.34(1)	1.371
0.96	2.09(4)	1.945	1.29(2)	1.246

S4 Sample code

Residue Interaction Diffusion Estimation in Perl:

```
#!/usr/bin/perl -w
"""
Created by Christopher J. Fennell
Updated and modified by Elham Fazelpour
Usage: input a pdb file and an area file to output the estimated
diffusion coefficient
ride.pl -options [pdb] [area]
"""
use Getopt::Std;
$temp = 310.15;
$viscosity = 0.000275;
$visc_correction = 0.398;
$ion_conc = 0;

$ACENME_coef = 0.054949348;

$GLY_coef = 0.0599;
$ALA_coef = 0.0718;
$SER_coef = 0.0677;
$PRO_coef = 0.0537;
$VAL_coef = 0.0504;
$THR_coef = 0.0595;
$CYS_coef = 0.0520;
$ILE_coef = 0.0530;
$LEU_coef = 0.0521;
$ASP_coef = 0.0699;
$ASN_coef = 0.0575;
$GLU_coef = 0.0661;
$GLN_coef = 0.0573;
$LYS_coef = 0.0543;
$MET_coef = 0.0490;
$HIS_coef = 0.0528;
$PHE_coef = 0.0477;
$ARG_coef = 0.0554;
$TYR_coef = 0.0555;
$TRP_coef = 0.0408;
$other_coef = 0.0582; # weighted average based on observed AA frequency

# get our options
getopts('acehmzi:t:v:');

# if we don't have a filename, drop to -h
$opt_h = 'true' if $#ARGV != 1;

# our option output
if ($opt_h){
    print "\nride.pl: Residue Interaction Diffusion Estimation. Estimates a\n";
    print "\tdiffusion coefficient from a PDB and MSMS area file.\n\n";
    print "usage: $0 [-options] [.pdb file] [.area file]\n\n";
    print "  -a: print per residue area values\n";
    print "  -c: only print a single number: the estimated D_0\n";
    print "  -e: only print a single number: the estimated expt. D\n";
```

```
    print "  -h: show this message\n";
    print "  -m: use monopeptide hydrodiffusivity parameters rather than default decapeptide\n";
    print "  -z: add zwitterionic contribution to the radius\n\n";
    print "  -i real : ion concentration in M (moles/L) - default: 0\n";
    print "  -t real : temperature in Kelvin - default: 310.15\n";
    die   "  -v real : viscosity of TIP3P in kg/(m•s) - default: estimate from T and [salt]\n\n";
}

if (defined($opt_m)){
    $opt_m = 1;

    # Set the coefficients to those from monopeptides
    $GLY_coef = 0.06102744;
    $ALA_coef = 0.07296574;
    $PRO_coef = 0.06459204;
    $CYS_coef = 0.07945528;
    $THR_coef = 0.07639375;
    $SER_coef = 0.09423633;
    $VAL_coef = 0.07886861;
    $ILE_coef = 0.07177586;
    $ASN_coef = 0.09165428;
    $GLN_coef = 0.07891862;
    $MET_coef = 0.07650982;
    $LEU_coef = 0.07970668;
    $PHE_coef = 0.07278305;
    $ASP_coef = 0.11777973;
    $HIS_coef = 0.08592650;
    $LYS_coef = 0.08397802;
    $GLU_coef = 0.11012820;
    $TYR_coef = 0.08044105;
    $TRP_coef = 0.07338015;
    $ARG_coef = 0.08183072;
    $other_coef = 0.082446321; # weighted average based on observed AA frequency
}

if (defined($opt_i)){
    if ($opt_i =~ /^[0-9]/) {
        $ion_conc = $opt_i;
    } else {
        die "\n\t-i value ($opt_i) is not a valid number\n\tPlease choose a value between 0 and 2\n\n";
    }
    if ($ion_conc < 0 || $ion_conc > 2){
        die "\n\t-i value ($opt_i) is not a valid number\n\tPlease choose a value between 0 and 2\n\n";
        }
}

if (defined($opt_t)){
    if ($opt_t =~ /^[0-9]/) {
        $temp = $opt_t;
    } else {
        die "\n\t-t value ($opt_t) is not a valid temperature\n\tPlease choose a value between 273.15 and 373.15\n\n";
    }
    if ($temp < 273.15 || $temp > 373.15){
        die "\n\t-t value ($opt_t) is not a valid temperature\n\tPlease choose a value between 273.15 and 373.15\n\n";
    }
}

if (defined($opt_v)){
    if ($opt_v =~ /^[0-9]/) {
```

```
        $viscosity = $opt_v;
    } else {
        die "\n\t-v value ($opt_v) is not a valid viscosity\n\tPlease choose a real value greater than 0.00001 (example: 0.00031)\n";
    }
    if ($viscosity < 0.00001){
        die "\n\t-v value ($opt_v) is not a valid viscosity\n\tPlease choose a real value greater than 0.00001 (example: 0.00031)\n";
    }
}

if (defined($opt_z)){
    $add_zwit = $opt_z;
    $zwit_val = 8.369;
}

$pdb_file_name = $ARGV[0];
$area_file_name = $ARGV[1];

open PDBIN, "$pdb_file_name" || die "\nError: can't open $pdb_file_name\n";
open AREAIN, "$area_file_name" || die "\nError: can't open $area_file_name\n";

while (<PDBIN>){
    $linecode = substr($_,0,6);
    if ($linecode eq 'HETATM' || $linecode eq 'ATOM  '){
        $residue = substr($_,17,3);
        push(@residue_array, $residue);
    }
}
close(PDBIN);

$count = 0;
while(<AREAIN>){
    @line = split;
    $area = $line[1];
    push(@area_array, $area) if $count != 0;
    $count++;
}
close(AREAIN);

if ($#area_array != $#residue_array){
    die "\nError: area_array and residue_area are not the same length!\n\n";
}

$ACENME_area = 0;
$ALA_area = 0;
$ARG_area = 0;
$ASN_area = 0;
$ASP_area = 0;
$CYS_area = 0;
$GLU_area = 0;
$GLN_area = 0;
$GLY_area = 0;
$HIS_area = 0;
$ILE_area = 0;
$LEU_area = 0;
$LYS_area = 0;
$MET_area = 0;
$PHE_area = 0;
$PRO_area = 0;
$SER_area = 0;
```

```
$THR_area = 0;
$TRP_area = 0;
$TYR_area = 0;
$VAL_area = 0;
$other_area = 0;

for ($i=0; $i<=$#area_array; $i++){
    if ($residue_array[$i] eq 'ALA'){
        $ALA_area += $area_array[$i];
    } elsif ($residue_array[$i] eq 'ARG'){
        $ARG_area += $area_array[$i];
    } elsif ($residue_array[$i] eq 'ASN'){
        $ASN_area += $area_array[$i];
    } elsif ($residue_array[$i] eq 'ASP'){
        $ASP_area += $area_array[$i];
    } elsif ($residue_array[$i] eq 'CYS'){
        $CYS_area += $area_array[$i];
    } elsif ($residue_array[$i] eq 'GLU'){
        $GLU_area += $area_array[$i];
    } elsif ($residue_array[$i] eq 'GLN'){
        $GLN_area += $area_array[$i];
    } elsif ($residue_array[$i] eq 'GLY'){
        $GLY_area += $area_array[$i];
    } elsif ($residue_array[$i] eq 'HIS'){
        $HIS_area += $area_array[$i];
    } elsif ($residue_array[$i] eq 'ILE'){
        $ILE_area += $area_array[$i];
    } elsif ($residue_array[$i] eq 'LEU'){
        $LEU_area += $area_array[$i];
    } elsif ($residue_array[$i] eq 'LYS'){
        $LYS_area += $area_array[$i];
    } elsif ($residue_array[$i] eq 'MET'){
        $MET_area += $area_array[$i];
    } elsif ($residue_array[$i] eq 'PHE'){
        $PHE_area += $area_array[$i];
    } elsif ($residue_array[$i] eq 'PRO'){
        $PRO_area += $area_array[$i];
    } elsif ($residue_array[$i] eq 'SER'){
        $SER_area += $area_array[$i];
    } elsif ($residue_array[$i] eq 'THR'){
        $THR_area += $area_array[$i];
    } elsif ($residue_array[$i] eq 'TRP'){
        $TRP_area += $area_array[$i];
    } elsif ($residue_array[$i] eq 'TYR'){
        $TYR_area += $area_array[$i];
    } elsif ($residue_array[$i] eq 'VAL'){
        $VAL_area += $area_array[$i];
    } elsif ($residue_array[$i] eq 'ACE' || $residue_array[$i] eq 'NME'){
        $ACENME_area += $area_array[$i];
    } else {
        $other_area += $area_array[$i];
    }
}

$stokes_sq = 0;
$stokes_sq += $ACENME_coef * $ACENME_area;
$stokes_sq += $ALA_coef * $ALA_area;
$stokes_sq += $ARG_coef * $ARG_area;
$stokes_sq += $ASN_coef * $ASN_area;
```

```
$stokes_sq += $ASP_coef * $ASP_area;
$stokes_sq += $CYS_coef * $CYS_area;
$stokes_sq += $GLU_coef * $GLU_area;
$stokes_sq += $GLN_coef * $GLN_area;
$stokes_sq += $GLY_coef * $GLY_area;
$stokes_sq += $HIS_coef * $HIS_area;
$stokes_sq += $ILE_coef * $ILE_area;
$stokes_sq += $LEU_coef * $LEU_area;
$stokes_sq += $LYS_coef * $LYS_area;
$stokes_sq += $MET_coef * $MET_area;
$stokes_sq += $PHE_coef * $PHE_area;
$stokes_sq += $PRO_coef * $PRO_area;
$stokes_sq += $SER_coef * $SER_area;
$stokes_sq += $THR_coef * $THR_area;
$stokes_sq += $TRP_coef * $TRP_area;
$stokes_sq += $TYR_coef * $TYR_area;
$stokes_sq += $VAL_coef * $VAL_area;
$stokes_sq += $other_coef * $other_area;
if ($add_zwit){
    $stokes_sq += $zwit_val;
}

$stokes_radius = sqrt($stokes_sq);

if (!defined($opt_v)){
    calculate_viscosity();
}

$diff_coef = ((1.3806485E-23*$temp)/(6*3.1415926536*$viscosity*$stokes_radius*1E-10))*1E9;
$visc_corrected_diff_coef = $diff_coef*$visc_correction;

if (defined($opt_a)){
    print "\nresidue areas (Å^2):\n";
}
if (defined($opt_a)){
    print "
ALA area:    $ALA_area
ARG area:    $ARG_area
ASN area:    $ASN_area
ASP area:    $ASP_area
CYS area:    $CYS_area
GLN area:    $GLN_area
GLU area:    $GLU_area
GLY area:    $GLY_area
HIS area:    $HIS_area
ILE area:    $ILE_area
LEU area:    $LEU_area
LYS area:    $LYS_area
MET area:    $MET_area
PHE area:    $PHE_area
PRO area:    $PRO_area
SER area:    $SER_area
THR area:    $THR_area
TRP area:    $TRP_area
TYR area:    $TYR_area
VAL area:    $VAL_area
ACENME area: $ACENME_area
Other area: $other_area\n";
```

```
}

if (!defined($opt_v) && !defined($opt_c) && !defined($opt_e)){
    printf "\nEst. Expt. viscosity:   %10.8f kg/(m•s)", $viscosity/$visc_correction;
    printf "\nEst. TIP3P viscosity:   %10.8f kg/(m•s)", $viscosity;
    printf "\nVisc. correction ratio: %10.6f\n", $visc_correction;
}

if (!defined($opt_c) && !defined($opt_e)){
printf "\nStokes radius: %12.6f Å\n", $stokes_radius;
printf "D_0:           %12.6f x 10^-5 cm^2/s\n", $diff_coef;
printf "D_v:           %12.6f x 10^-5 cm^2/s\n\n", $visc_corrected_diff_coef;
} elsif (defined($opt_c)){
printf "%12.6f\n", $diff_coef;
} elsif (defined($opt_e)) {
printf "%12.6f\n", $visc_corrected_diff_coef;
} else {
    print "\nHmmm... Don't know what to print...\n\n";
}


sub calculate_viscosity{
    $ratio_293 = 0.3363;
    $ratio_298 = 0.3584;
    $ratio_310 = 0.398;
    $temp_cent = $temp - 273.15;
    $expt_viscosity = 0.00003272*$temp_cent**4 - 0.0091*$temp_cent**3 + 0.9928*$temp_cent**2 - 55.641*$temp_cent + 1786.8;
    $expt_viscosity *= 1E-3;
    $visc_ratio = $expt_viscosity / 1.002;
    #$salt_viscosity = 0.0033*$ion_conc**3-0.0009*$ion_conc**2+0.0957*$ion_conc+1.002;
    $salt_viscosity = (0.0359*$ion_conc**2+0.0669*$ion_conc+0.3395)/$ratio_293;

    $expt_viscosity = ($visc_ratio * $salt_viscosity)*1E-3;

    # find the viscosity correction
    # currently, we assume a linear relation between known ratios for TIP3P to Expt.
    $vc_slope = ($ratio_310 - $ratio_298)/12;
    $visc_correction = ($temp - 298.15)*$vc_slope + $ratio_298;

    $viscosity = $expt_viscosity * $visc_correction;
}
```

Residue Interaction Diffusion Estimation in Python :

```
#!/bin/bash/env python

""" ride tool in python
Author: Christopher J. Fennell
Python vesrion Elham Fazelpour (Spring 2021)
"""
######################
import os
import sys
import math
import numpy as np
import argparse
######################
parser = argparse.ArgumentParser(description="ride.py: Residue Interaction Diffusion Estimation. Estimates a diffusion coefficient from a PDB and MSMS area file.")

parser.add_argument("-a", action="store_true", help="print per residue area values")
parser.add_argument("-c", action="store_true", help="only print a single number: the estimated D_0")
parser.add_argument("-e", action="store_true", help="only print a single number: the estimated expt. D")
parser.add_argument("-help",action="store_true", help="show this message")
parser.add_argument("-i", type=float, default=0, help="ion concentration in M (moles/L) - default: 0")
parser.add_argument("-t", type=float, default=310.15, help="temperature in Kelvin - default: 310.15")
parser.add_argument("-v", type=float, help="viscosity of TIP3P in kg/(m.s) - default: estimate from T and [salt]")
parser.add_argument("pdbfile", help="path to PDB file")
parser.add_argument("areafile", help="path to MSMS area file")

args = parser.parse_args()

if args.help:
    parser.print_help()
    exit()

##### default values
temp = 310.15
viscosity = 0.0002749
visc_correction = 0.39768
ion_conc = 0
ACENME_coef= 0.05701
pdbfile = args.pdbfile
areafile = args.areafile
##############################

def calculate_viscosity(temp=args.t, ion_conc=args.i):
    temp_cent = temp - 273.15
    expt_viscosity = -4.25723E-7*temp_cent**5+0.000139219*temp_cent**4 - 0.0184566*temp_cent**3 + 1.32521096*temp_cent**2-59.69364345*temp_cent + 1790.0825625
    expt_viscosity *= 1E-3
    visc_ratio = expt_viscosity / 1.00242
    salt_viscosity = -0.00239*ion_conc**4+ 0.01723* ion_conc**3 - 0.02234*ion_conc**2+ 0.10201*ion_conc +1.00242
    expt_viscosity = (visc_ratio * salt_viscosity) * 1E-3

    # find the viscosity correction
    # currently, we assume a linear relation between known ratios for TIP3P to Expt.
    ratio_298 = 0.357938
    ratio_310 = 0.397681
    vc_slope = (ratio_310 - ratio_298) / 12
    visc_correction = (temp - 298.15) * vc_slope + ratio_298
```

```
    viscosity = expt_viscosity * visc_correction
    return viscosity

###### aa-coef, decamer

GLY_coef= 0.05996
ALA_coef= 0.07184
PRO_coef= 0.05374
CYS_coef= 0.05200
THR_coef= 0.05958
SER_coef= 0.06774
VAL_coef= 0.05039
ILE_coef= 0.05304
ASN_coef= 0.05756
GLN_coef= 0.05734
MET_coef= 0.04901
LEU_coef= 0.05210
PHE_coef= 0.04769
ASP_coef= 0.06998
HIS_coef= 0.05279
LYS_coef= 0.05426
GLU_coef= 0.06617
TYR_coef= 0.05553
TRP_coef= 0.04087
ARG_coef= 0.05548
# weighted average based on observed AA frequency
other_coef = 0.05822
############# check the input options (ionic concentration, temperature and viscosity)

if args.i:
    try:
        ion_conc = float(args.i)
        if ion_conc < 0 or ion_conc > 2:
            raise ValueError
    except ValueError:
        print("\n\t-i value ({}) is not a valid number\n\tPlease choose a value between 0 and 2\n\n".format(args.i))
        exit()

if args.t is not None:
    # if args.t.isdigit():
    temp = int(args.t)
    # else:
    #   raise ValueError(f'\n\t-t value ({args.t}) is not a valid temperature \n\t Please choose a value between 273.15 and 373.15\n\n')

    if temp < 273.15 or temp > 373.15:
        raise ValueError(f'\n\t-t value ({args.t}) is not a valid temperature \n\t Please choose a value between 273.15 and 373.15\n\n')



if args.v is not None:
    if args.v.isdigit():
        viscosity = float(args.v)
    else:
        raise ValueError(f"\n\t-v value ({args.v}) is not a valid viscosity\n\tPlease
         choose a real value greater than 0.00001 (example: 0.00031)\n")
    if viscosity < 0.00001:
        raise ValueError(f"\n\t-v value ({args.v}) is not a valid viscosity\n\tPlease
        choose a real value greater than 0.00001 (example: 0.00031)\n")
```

```
with open(pdbfile, 'r') as pdbin:
    lines=pdbin.readlines()
    residue_array=[]
    for line in lines:
        linecode = line[0:6]
        if linecode == 'HETATM' or linecode == 'ATOM  ':
            residue_array += line[17:20].split()



with open(areafile, 'r') as areain:
    count = 0
    area_array=[]
    for line in areain:
        line = line.strip()
        if count != 0:
            area = float(line.split()[1])
            area_array.append(area)
        count += 1

if len(area_array) != len(residue_array):
    raise ValueError("\nError: area_array and residue_array are not the same length!\n\n")

ACENME_area = 0
ALA_area = 0
ARG_area = 0
ASN_area = 0
ASP_area = 0
CYS_area = 0
GLU_area = 0
GLN_area = 0
GLY_area = 0
HIS_area = 0
ILE_area = 0
LEU_area = 0
LYS_area = 0
MET_area = 0
PHE_area = 0
PRO_area = 0
SER_area = 0
THR_area = 0
TRP_area = 0
TYR_area = 0
VAL_area = 0
other_area = 0

for i in range(len(area_array)):
    if residue_array[i] =='ALA':
        ALA_area += area_array[i]
    elif residue_array[i] =='ARG':
        ARG_area += area_array[i]
    elif residue_array[i] =='ASN':
        ASN_area += area_array[i]
    elif residue_array[i] =='ASP':
        ASP_area += area_array[i]
    elif residue_array[i] =='CYS':
        CYS_area += area_array[i]
    elif residue_array[i] =='GLU':
```

```
        GLU_area += area_array[i]
    elif residue_array[i] =='GLN':
        GLN_area += area_array[i]
    elif residue_array[i] =='GLY':
        GLY_area += area_array[i]
    elif residue_array[i] =='HIS':
        HIS_area += area_array[i]
    elif residue_array[i] == 'ILE':
        ILE_area += area_array[i]
    elif residue_array[i] == 'LEU':
        LEU_area += area_array[i]
    elif residue_array[i] == 'LYS':
        LYS_area += area_array[i]
    elif residue_array[i] == 'MET':
        MET_area += area_array[i]
    elif residue_array[i] == 'PHE':
        PHE_area += area_array[i]
    elif residue_array[i] == 'PRO':
        PRO_area += area_array[i]
    elif residue_array[i] == 'SER':
        SER_area += area_array[i]
    elif residue_array[i] == 'THR':
        THR_area += area_array[i]
    elif residue_array[i] == 'TRP':
        TRP_area += area_array[i]
    elif residue_array[i] == 'TYR':
        TYR_area += area_array[i]
    elif residue_array[i] == 'VAL':
        VAL_area += area_array[i]
    else:
        other_area += area_array[i]

stokes_sq = 0
stokes_sq += ACENME_coef * ACENME_area
stokes_sq += ALA_coef * ALA_area
stokes_sq += ARG_coef * ARG_area
stokes_sq += ASN_coef * ASN_area
stokes_sq += ASP_coef * ASP_area
stokes_sq += CYS_coef * CYS_area
stokes_sq += GLU_coef * GLU_area
stokes_sq += GLN_coef * GLN_area
stokes_sq += GLY_coef * GLY_area
stokes_sq += HIS_coef * HIS_area
stokes_sq += ILE_coef * ILE_area
stokes_sq += LEU_coef * LEU_area
stokes_sq += LYS_coef * LYS_area
stokes_sq += LYN_coef * LYN_area
stokes_sq += MET_coef * MET_area
stokes_sq += PHE_coef * PHE_area
stokes_sq += PRO_coef * PRO_area
stokes_sq += SER_coef * SER_area
stokes_sq += THR_coef * THR_area
stokes_sq += TRP_coef * TRP_area
stokes_sq += TYR_coef * TYR_area
stokes_sq += VAL_coef * VAL_area
stokes_sq += other_coef * other_area

stokes_radius = math.sqrt(stokes_sq)
print(stokes_radius)
```

```
if args.v is None:
    calculate_viscosity()

diff_coef = ((1.3806485E-23*temp)/
(6*3.1415926536*viscosity*stokes_radius*1E-10))*1E9
visc_corrected_diff_coef = diff_coef*visc_correction

if args.a is not None:
    print("\nresidue areas (Å^2):\n")
if args.a is not None:
    print(f"""
ALA area:    {ALA_area}
ARG area:    {ARG_area}
ASN area:    {ASN_area}
ASP area:    {ASP_area}
CYS area:    {CYS_area}
GLN area:    {GLN_area}
GLU area:    {GLU_area}
GLY area:    {GLY_area}
HIS area:    {HIS_area}
ILE area:    {ILE_area}
LEU area:    {LEU_area}
LYS area:    {LYS_area}
MET area:    {MET_area}
PHE area:    {PHE_area}
PRO area:    {PRO_area}
SER area:    {SER_area}
THR area:    {THR_area}
TRP area:    {TRP_area}
TYR area:    {TYR_area}
VAL area:    {VAL_area}
ACENME area: {ACENME_area}
Other area: {other_area}\n""")

if args.v is not None and args.c is not None and args.e is not None:
    print(f"\nEst. Expt. viscosity:   {viscosity/visc_correction:.8f} kg/(m•s)")
    print(f"Est. TIP3P viscosity:   {viscosity:.8f} kg/(m•s)")
    print(f"Visc. correction ratio: {visc_correction:.6f}\n")

if args.c is not None and args.e is not None:
    print(f"Stokes radius: {stokes_radius:.6f} Å")
    print(f"D_0:           {diff_coef:.6f} x 10^-5 cm^2/s")
    print(f"D_v:           {visc_corrected_diff_coef:.6f} x 10^-5 cm^2/s\n")

elif args.c is not None:
    print(f"{diff_coef:.6f}")

elif args.e is not None:
    print(f"{visc_corrected_diff_coef:.6f}")

else:
    print("\nHmmm... Don’t know what to print...\n\n")
```

References

(1) Hess, B. Determining the shear viscosity of model liquids from molecular dynamics simulations. *The Journal of Chemical Physics* **2002**, *116*, 209–217.

(2) Zwanzig, R. Time-Correlation Functions and Transport Coefficients in Statistical Mechanics. *Annual Review of Physical Chemistry* **1965**, *16*, 67–102.

(3) Brenner, H. Coupling between the translational and rotational brownian motions of rigid particles of arbitrary shape I. Helicoidally isotropic particles. *Journal of Colloid Science* **1965**, *20*, 104–122.

(4) Brenner, H. Coupling between the translational and rotational brownian motions of rigid particles of arbitrary shape: II. General theory. *Journal of Colloid and Interface Science* **1967**, *23*, 407–436.

(5) Kabsch, W.; Sander, C. Dictionary of protein secondary structure: Pattern recognition of hydrogen-bonded and geometrical features. *Biopolymers* **1983**, *22*, 2577–2637.

(6) Zhang, H.; Yin, C.; Jiang, Y.; van der Spoel, D. Force field benchmark of amino acids: I. hydration and diffusion in different water models. *Journal of Chemical Information and Modeling* **2018**, *58*, 1037–1052.

(7) Germann, M. W.; Turner, T.; Allison, S. A. Translational Diffusion Constants of the Amino Acids: Measurement by NMR and Their Use in Modeling the Transport of Peptides. *The Journal of Physical Chemistry A* **2007**, *111*, 1452–1455.

(8) Yeh, I.-C.; Hummer, G. Diffusion and Electrophoretic Mobility of Single-Stranded RNA from Molecular Dynamics Simulations. *Biophysical Journal* **2004**, *86*, 681–689.
